# HIV-1 infection depletes human CD34^+^CD38^-^ hematopoietic progenitor cells via pDC-dependent mechanisms

**DOI:** 10.1371/journal.ppat.1006505

**Published:** 2017-07-31

**Authors:** Guangming Li, Juanjuan Zhao, Liang Cheng, Qi Jiang, Sheng Kan, Enqiang Qin, Bo Tu, Xin Zhang, Liguo Zhang, Lishan Su, Zheng Zhang

**Affiliations:** 1 The Lineberger Comprehensive Cancer Center, University of North Carolina, Chapel Hill, NC, United States of America; 2 Research Center for Clinical & Translational Medicine, Beijing 302 Hospital, Beijing, China; 3 Treatment and Research Center for Infectious Diseases, Beijing 302 Hospital, Beijing, China; 4 Key laboratory of Infection and Immunity, Institute of Biophysics, Chinese Academy of Science, Beijing, China; Emory University, UNITED STATES

## Abstract

Chronic human immunodeficiency virus-1 (HIV-1) infection in patients leads to multi-lineage hematopoietic abnormalities or pancytopenia. The deficiency in hematopoietic progenitor cells (HPCs) induced by HIV-1 infection has been proposed, but the relevant mechanisms are poorly understood. We report here that both human CD34^+^CD38^-^ early and CD34^+^CD38^+^ intermediate HPCs were maintained in the bone marrow (BM) of humanized mice. Chronic HIV-1 infection preferentially depleted CD34^+^CD38^-^ early HPCs in the BM and reduced their proliferation potential *in vivo* in both HIV-1-infected patients and humanized mice, while CD34^+^CD38^+^ intermediate HSCs were relatively unaffected. Strikingly, depletion of plasmacytoid dendritic cells (pDCs) prevented human CD34^+^CD38^-^ early HPCs from HIV-1 infection-induced depletion and functional impairment and restored the gene expression profile of purified CD34^+^ HPCs in humanized mice. These findings suggest that pDCs contribute to the early hematopoietic suppression induced by chronic HIV-1 infection and provide a novel therapeutic target for the hematopoiesis suppression in HIV-1 patients.

## Author summary

Multi-lineage hematopoietic abnormalities generally occur during chronic infection which results in a disorder of human leukocyte development and differentiation, contributing to human immunodeficiency virus-1 (HIV-1)-infection induced immune-pathogenesis in AIDS patients. Although successful antiretroviral therapy can reduce plasma viral loads to undetectable levels and ameliorate HIV-1-associated hemato-suppression, immune cell development is only partially restored. The mechanism for the abnormal hematopoiesis occurring during chronic HIV-1 infection remains unclear. HIV-1 infection may directly or indirectly functionally impair hematopoietic progenitors by either viral products or induction of persistent inflammatory responses, leading to hematopoiesis obstacles. Here, we show that HIV-1 infection significantly depleted and functionally impaired human hematopoietic progenitors in the bone marrow of both HIV-1-infected patients and humanized mice through a plasmacytoid dendritic cell (pDC)-dependent mechanism, as depletion of pDCs significantly recovered cell numbers and functions and gene expression profiles of hematopoietic progenitor cells in humanized mice *in vivo*. Our study clarifies a novel mechanism underlying hemato-suppression induced by chronic HIV-1 infection and provides a novel strategy to halt HIV-1 disease.

## Introduction

Human immunodeficiency virus-1 (HIV-1) infection in patients leads to multi-lineage hematopoietic abnormalities, including anemia, granulocytopenia and thrombocytopenia [1,2]. Abnormalities in fetal hematopoiesis have also been reported in aborted fetuses from HIV-1 seropositive women [3]. The defect in hematopoietic progenitor cells (HPCs) or hematopoiesis induced by HIV-1 has been proposed [1,4,5]. In addition, the degree of the hematopoietic pathology correlates with the stage of disease progression [6], and end-stage disease is characterized by pancytopenia [1]. Long-term bone marrow (BM) cultures from HIV-1-infected patients exhibit low CD34^+^ progenitor cell growth and differentiation [7,8], indicating functional impairment of early hematopoietic progenitors. Although the successful highly active antiretroviral therapy (HAART) clearly ameliorates HIV-1-associated hemato-suppression, it does not completely restore blood cell development [9]. These observations indicate that hematopoietic failure is an important aspect of HIV-1 infection-induced pathogenesis [10].

HPCs are comprised of diverse populations, including both early and intermediate progenitors. Each subpopulation expresses distinct sets of cell surface antigens, although they all express the cell surface antigen CD34 [11,12]. Early and intermediate populations can be distinguished by the expression of CD38, with the former being negative for CD38 and the latter being positive for this antigen. Functionally, intermediate progenitors include common myeloid progenitors that can give rise to all myeloid, erythroid and megakaryocyte lineages. Due to limited access to the BM in humans, properties of human HPC subsets and their alterations in healthy and HIV-1 disease states have been difficult to characterize.

The mechanisms underlying abnormal hematopoiesis in HIV-1 infection remains unclear due to the paucity of robust animal models that mimic human hemato-suppression *in vivo*. Although previous studies failed to detect HIV-1 infection of HPCs, recent reports indicated that HIV-1 could directly infect HPC subsets and lead to their impairment [13–16]. In addition, HIV-1 proteins such as Nef [17] and prolonged treatment with antiretroviral drugs could also compromise hematopoietic progenitors [18]. Although these studies investigated a litany of direct and indirect causes of HIV-1-associated hemato-suppression, how HIV-1 affects hematopoiesis *in vivo* remains unclear.

There is emerging evidence that certain cytokines induced during inflammation have significant effects on HPCs in the BM. Type I and II interferon (IFN) [19–23], tumor necrosis factor (TNF) [24–26] and lipopolysaccharide (LPS) [27,28] directly stimulate HPC proliferation and differentiation, thereby increasing the short-term output of mature effector leukocytes. However, chronic inflammatory cytokine signaling can lead to functional exhaustion of HPCs [19,22,28]. Our previous study demonstrated that plasmacytoid dendritic cells (pDCs), the major type I interferon (IFN-I)-producing cells during acute or chronic HIV-1 infection, could inhibit viral replication while significantly contributing to HIV-1 infection-induced immune-pathogenesis, including increased immune cell death and reduced immune reconstitution of human CD45^+^ cells in humanized mice *in vivo* [29]. These findings suggest that pDCs play a pivotal role in the hemato-suppression induced by chronic HIV-1 infection.

In this study, we sought to understand the role of pDCs in HIV-1-associated hemato-suppression in a humanized mouse model *in vivo*. We discovered that HIV-1 infection depleted CD34^+^CD38^-^ early HPCs and functionally impaired human CD34^+^ HPCs in the BM of patients and humanized mice with HIV-1 infection. This phenomenon was further found to be dependent on pDCs, as depletion of pDCs significantly recovered HPC cell numbers and multi-lineage colony-forming functions. Our present study therefore reveals a novel mechanism for hematopoiesis suppression induced by chronic HIV-1 infection and provides a new strategy to rescue HPC function and halt HIV-1 disease progression.

## Results

### Development and maintenance of functional human HPCs in humanized mice

**Fig 1:**
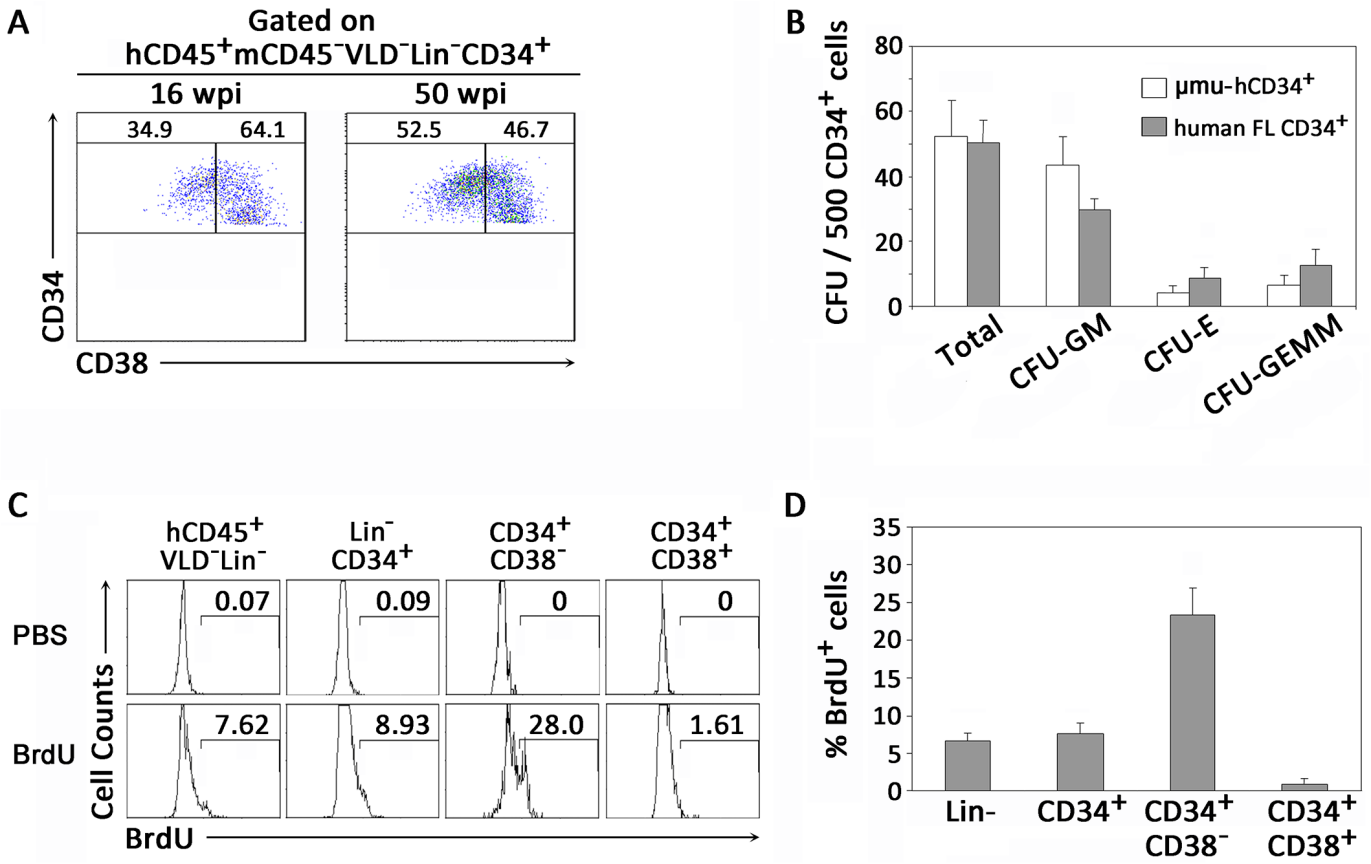
Development of functional human CD34^+^ HPCs in BM of humanized mice. (A) Representative dot plots showing distribution of human early CD34^+^CD38^-^ and intermediate CD34^+^CD38^+^ HPCs in BM of humanized mice. Numbers indicate percentages of CD34^+^CD38^-^ and CD34^+^CD38^+^ HPCs (gated on hCD45^+^mCD45^-^VLD^-^Lin^-^CD34^+^ cells). Data are representative of two independent experiments with mice reconstituted with three human donors. (B) Pooled data indicating numbers of colonies that developed from human CD34^+^ HPCs from BM of humanized mice and human fetal livers (n = 6). Total colonies, CFU-GM, colony-forming unit-granulocyte, macrophage; CFU-E, colony-forming unit-erythroid; CFU-GEMM, colony-forming unit-granulocyte, erythroid, macrophage, megakaryocyte. (C) Representative histograms showing proliferation of CD34^+^ HPCs *in vivo* through BrdU labeling in humanized mice. PBS mouse samples were used as controls (upper panels). Numbers indicate percentages of BrdU^+^ HPCs within CD34^+^ HPC subsets. (D) Pooled data indicate percentages of BrdU^+^ CD34^+^ HPC subsets in BM of humanized mice (n = 5). (B, D) Data are shown as the mean ± s.e.m.

By gating on live human CD45^+^ cells (VLD^-^mCD45^-^) with a lymphoid morphology that lacked common markers (Lineage^−^) for T cells (CD3), B cells (CD19 and CD20) or NK cells (CD56 and CD16), we identified human BM-derived HPCs as CD34^+^ cells, which included early CD34^+^CD38^-^ and intermediate CD34^+^CD38^+^ subpopulations (S1 Fig). We then analyzed the human HPCs from the BM of humanized mice at various time points after human CD34^+^ cell transplantation. The early and intermediate HPCs could be detected significantly at both 16 weeks and 50 weeks after CD34^+^ cell transplantation with relatively stable levels (Fig 1A).

To determine if human HPCs derived from humanized mice were functional, human CD34^+^ cells were isolated from the BM of humanized mice and human fetal livers, and colony-forming unit (CFU) assays were performed by culturing CD34^+^ HPCs in a complete methylcellulose medium system. Two weeks later, HPCs derived from the BM of humanized mice produced 50 colonies on average for every 500 CD34^+^ cells plated, similar to that of human fetal liver-derived CD34^+^ cells (Fig 1B). All hematopoietic lineages were generated in cultures from the BM of humanized mice. In addition, HPCs from humanized mice could proliferate and differentiate into various blood lineage cells *in vitro* at a similar frequency to that of CD34^+^ cells from human fetal livers, including colony-forming unit-granulocyte and macrophage (CFU-GM), colony-forming unit-erythroid (CFU-E) and colony-forming unit-granulocyte, erythroid, macrophage, megakaryocyte (CFU-GEMM) (Fig 1B). We then measured the proliferation capacity of HPCs by BrdU labeling *in vivo* and found that 8.9% of human CD34^+^ cells showed proliferation (Fig 1C). Notably, the CD34^+^CD38^-^ early HPCs were much more proliferative, with an average of nearly 25% of cells being BrdU positive, which was significantly higher than the relatively quiescent CD34^+^CD38^+^ intermediate HPCs with 1.6% BrdU labeling (Fig 1C and 1D). These data suggest that the human CD34^+^CD38^-^ early HPCs and CD34^+^CD38^+^ intermediate HPCs were both functionally developed and maintained in the BM of humanized mice.

### CD34^+^CD38^-^ early HPCs are preferentially depleted *in vivo* in both HIV-1 chronically infected patients and humanized mice

**Fig 2:**
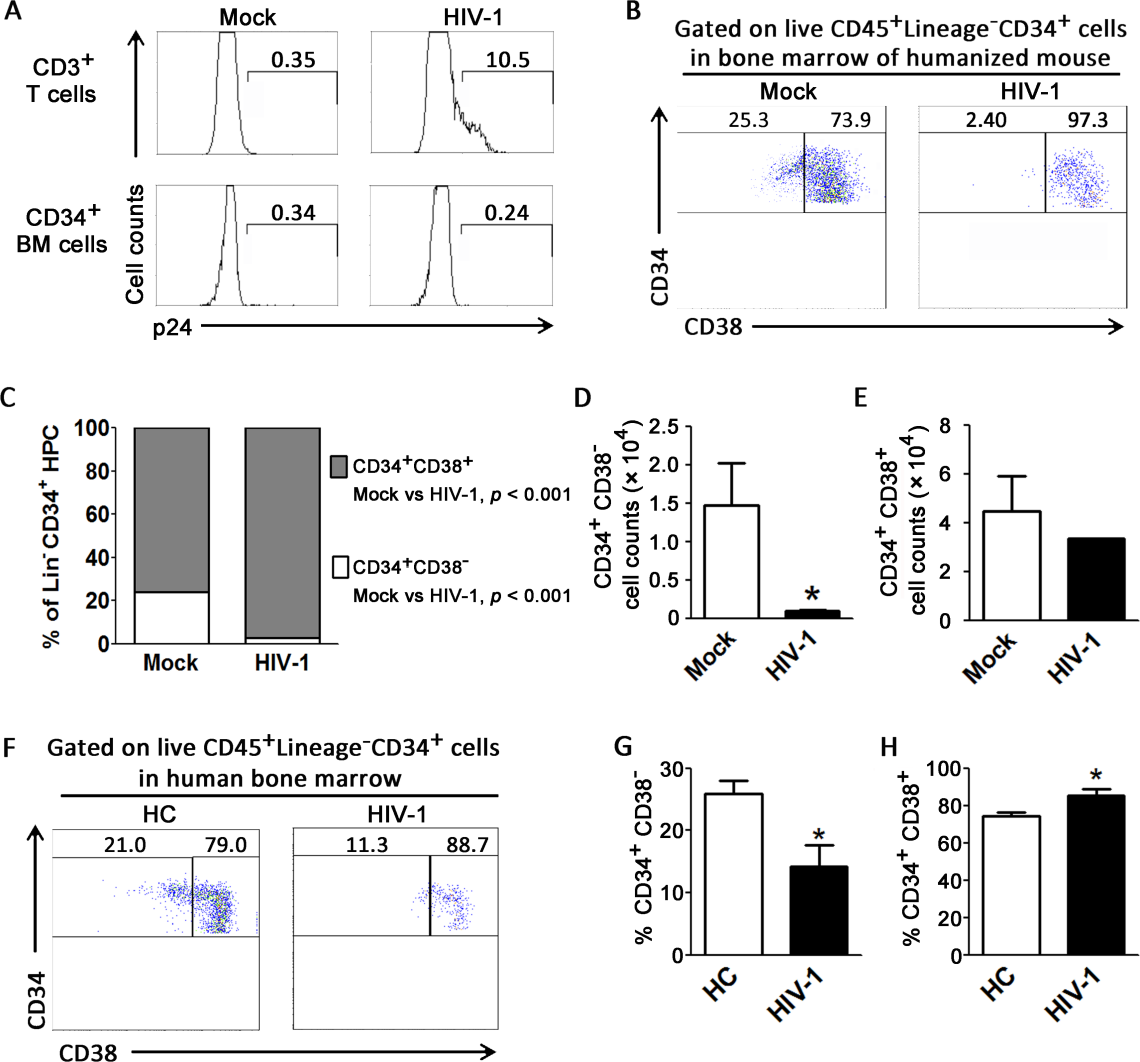
Chronic HIV-1 infection preferentially depletes early CD34^+^CD38^-^ HPCs in humanized mice *in vivo*. (A) Representative histograms showing p24 expression on CD3^+^ T cells and CD34^+^ HPCs from BM of humanized mice with chronic HIV-1 infection. Numbers indicate percentages of p24-expressing CD3^+^ T cells and CD34^+^ HPCs. Data are representative of three independent experiments with mice reconstituted with two to three donors. (B) Representative dot plots showing chronic HIV-1 infection depleting early CD34^+^CD38^-^ HPCs from BM of humanized mice. Numbers indicate percentages of early CD38^-^CD34^+^ and intermediate CD34^+^CD38^+^ HPCs. (C) Pooled data indicating mean percentages of early CD34^+^CD38^-^ HPCs and intermediate CD34^+^CD38^+^ HPCs in BM of humanized mice with mock and HIV-1 infection shown in the stacked bar graph. *P* values are shown. (D-E) Pooled data indicating absolute cell numbers of early CD34^+^CD38^-^ HPCs (D) and intermediate CD34^+^CD38^+^ HPCs (E) in BM of humanized mice with mock (n = 7) or chronic HIV-1 infection (n = 5). (F) Representative dot plots showing chronic HIV-1 infection depleting early CD34^+^CD38^-^ HPCs from BM of HIV-1-infected patients compared to that of healthy control (HC) donors. Numbers indicate percentages of early CD38^-^CD34^+^ and intermediate CD34^+^CD38^+^ HPCs. (G-H) Pooled data indicating percentages of early CD34^+^CD38^-^ HPCs (G) and intermediate CD34^+^CD38^+^ HPCs (H) in BM of HC donors (n = 6) and HIV-1-infected patients (n = 5). (D, E, G, H) Data are shown as the mean ± s.e.m. *P < 0.05 and ***P < 0.001 (two-tailed unpaired Student’s t-test).

Utilizing the robust animal model, we were able to investigate whether chronic HIV-1 infection affected human HPCs. HIV-1 infection was established in humanized mice, as measured by plasma HIV-1 RNA (copies/mL, S2 Fig). On termination, we also measured HIV-1 gag p24 expression in both T cells and CD34^+^ HPCs by flow cytometry. Although a previous study suggested that HIV-1 has the potential to infect intermediate CD34^+^CD38^+^ HPCs [13], we found that p24 expression was absent in BM CD34^+^ HPCs from humanized mice with HIV-1 infection; in contrast, CD3^+^ T cells showed high levels of p24 expression (10.5%) (Fig 2A). Further analysis indicated that the frequency of CD34^+^CD38^-^ early HPCs was largely decreased in humanized mice with chronic HIV-1 infection, while the proportion of intermediate CD34^+^CD38^+^ HSCs was relatively expanded (Fig 2B).

Summarized data further demonstrated that chronic HIV-1 infection significantly reduced CD34^+^CD38^-^ early HPCs by nearly 8-fold as compared to the non-infected animals; meanwhile, the proportion of CD34^+^CD38^+^ intermediate HPCs was increased from 76% to nearly 100% as shown in the stacked bar graph (Fig 2C). When the absolute cell counts of early and intermediate HPCs were calculated, the number of CD34^+^CD38^-^ early HPCs was dramatically reduced in the BM of chronically infected animals (Fig 2D), while CD34^+^CD38^+^ intermediate HPC counts were affected mildly (Fig 2E). These results suggest a depletion of early HPCs during chronic HIV-1 infection. Importantly, we observed a similar depletion of BM CD34^+^CD38^-^ early HPCs in HIV-1-infected patients. As shown in Fig 2F, the percentage of CD34^+^CD38^-^ early HPCs was significantly decreased within total CD34^+^ HPCs in an HIV-1-infected patient when compared to a healthy control (HC). The depletion of CD34^+^CD38^-^ early HPCs in human BM from HIV-1 infection was strengthened by the addition of more patients (n = 5) (Fig 2G). In contrast, the proportion of intermediate CD34^+^CD38^+^ HSCs was increased within total HPCs in HIV-1-infected patients relative to those of HC subjects (Fig 2H). These data indicated that early CD34^+^CD38^-^ HSCs were preferentially depleted by chronic HIV-1 infection, and the humanized mouse is a highly relevant animal model that mimics HIV-1-induced hemato-suppression conditions in patients.

### Chronic HIV-1 infection inhibits proliferation of human HPCs *in vivo*

**Fig 3:**
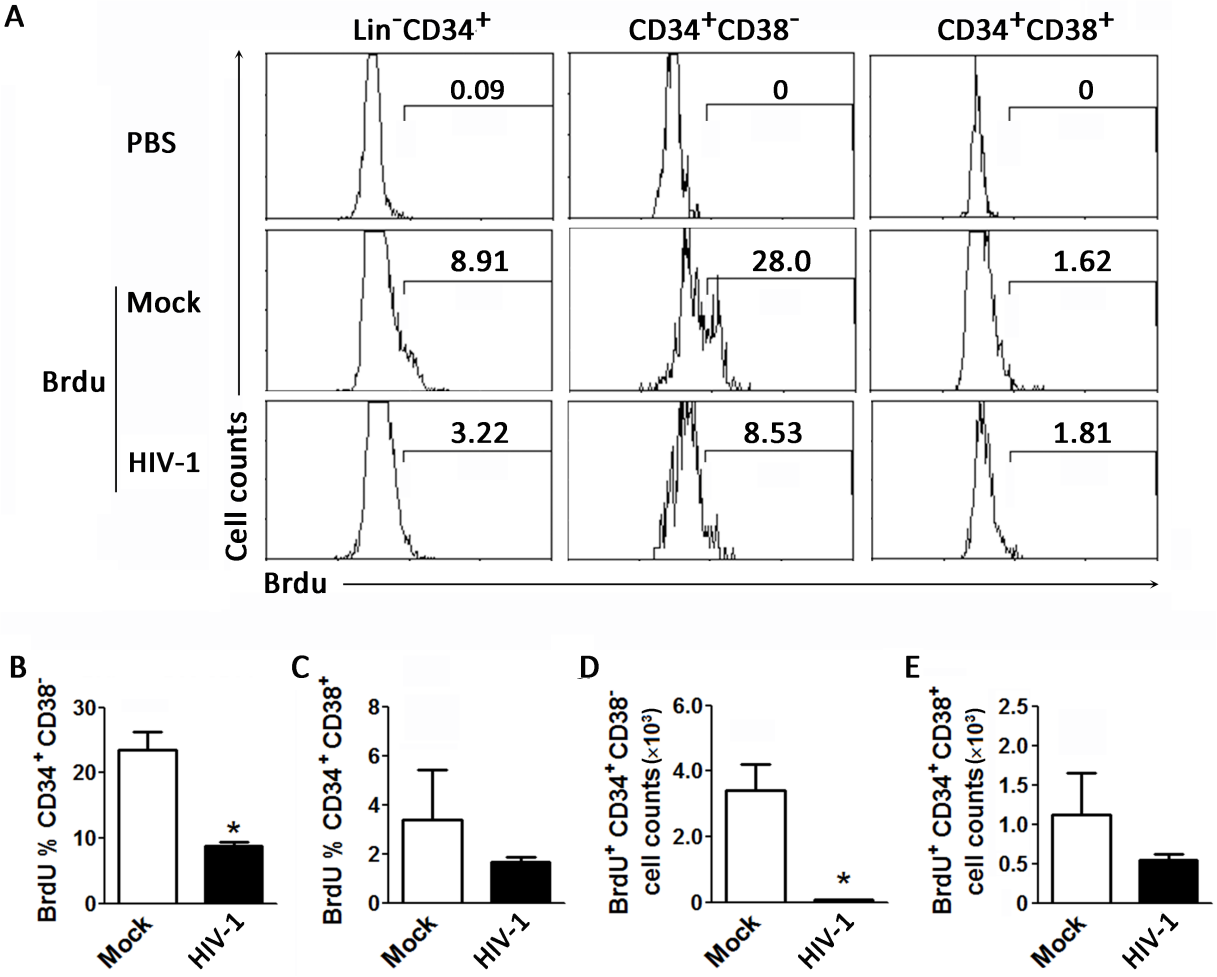
Chronic HIV-1 infection impairs proliferation of early CD34^+^CD38^-^ HPCs *in vivo* of humanized mice. (A) Representative histograms showing BrdU labeling of CD34^+^ HPC subsets from BM of humanized mice. Numbers indicate percentages of BrdU^+^ within the total CD34^+^ HPCs, early CD34^+^CD38^-^ HPCs and intermediate CD34^+^CD38^+^ HPCs. (B-E) Pooled data indicating percentages of BrdU^+^ cells within early CD34^+^CD38^-^ HPCs (B) and intermediate CD34^+^CD38^+^ HPCs (C); and absolute cell numbers of BrdU^+^ early CD34^+^CD38^-^ HPCs (D) and intermediate CD34^+^CD38^+^ HPCs (E) in BM of mock (n = 4) and HIV-1-infected (n = 6) humanized mice. Data are shown as the mean ± s.e.m. *P < 0.05 (two-tailed unpaired Student’s t-test).

We next analyzed the effect of chronic HIV-1 infection on the homeostatic proliferation of human HPCs in humanized mice. The results indicate that proliferation of human CD34^+^ cells in the BM was inhibited by approximately 3-fold in chronic HIV-1 infection compared to the mock animals (Fig 3A). Consistent with the preferential reduction of CD34^+^CD38^-^ HPCs, BrdU-positive CD34^+^CD38^-^ early HPCs were significantly decreased by chronic HIV-1 infection; meanwhile, the proliferation of CD34^+^CD38^+^ intermediate HPCs was only mildly reduced by chronic HIV-1 infection (Fig 3B and 3C). In terms of cell numbers, the early HPC counts were significantly decreased by chronic HIV-1 infection compared with mock treatment in mice (Fig 3D), while intermediate HPC counts were only slightly reduced (Fig 3E). Thus, while both cell types were less proliferative in the presence of HIV-1, the more marked difference was observed in CD34^+^CD38^-^ cells. Therefore, chronic HIV-1 infection appeared to suppress human hematopoiesis by inhibiting homeostatic proliferation of human early HPCs in the BM.

### Human CD34^+^ HPCs isolated from BM of mice chronically infected with HIV-1 display impaired differentiation

**Fig 4:**
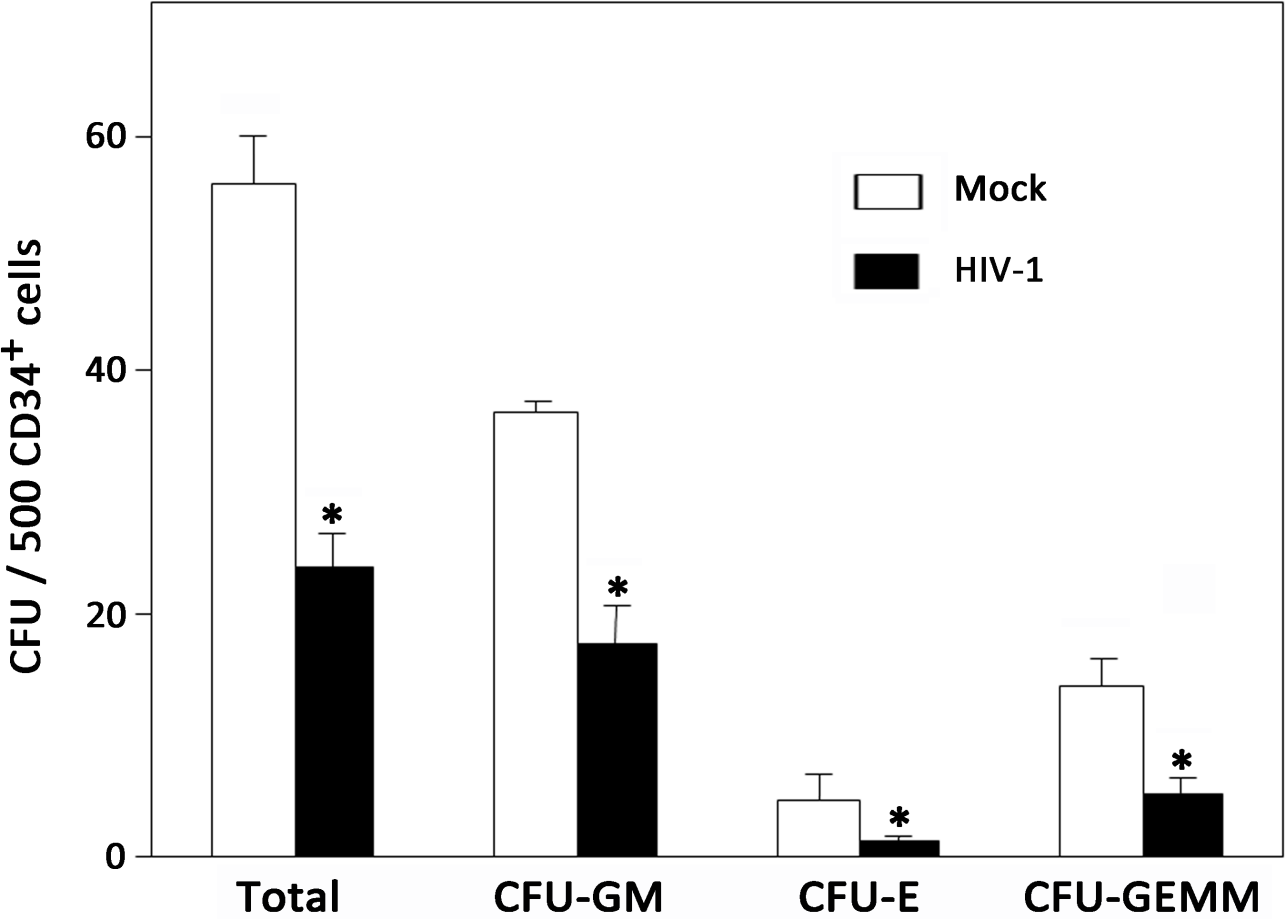
Chronic HIV-1 infection impairs colony-forming activity of CD34^+^ HPCs in BM of humanized mice. Pooled data indicate the number of colonies that developed from human CD34^+^ HPCs of mock (n = 4) and HIV-1-infected (n = 5) mice. Data are shown as the mean and s.e.m. *P < 0.05 (two-tailed unpaired Student’s t-test).

In order to assess the quality of *in vivo* human HPCs during chronic HIV-1 infection, we measured CFU activity of purified human Lin^-^CD34^+^ HPCs from mock- or HIV-1-infected humanized mice, including GM, E and GEMM (S3 Fig). As shown in Fig 4, HPCs isolated from uninfected mice consistently produced over 50 colonies per 500 CD34^+^ cells on average, whereas HPCs derived from HIV-1-infected mice produced less than 30 colonies per 500 CD34^+^ HPCs. Moreover, the ability of HPCs to generate all lineages, including GM, E and GEMM, was also suppressed to some extent in chronically infected mice. Therefore, the results indicated that chronic HIV-1 infection leads to the impaired differentiation of HPCs *in vivo*.

### Decreased quantity and quality of CD34^+^CD38^-^ early HPCs is dependent on pDCs during chronic HIV-1 infection

Increasing reports have demonstrated that chronic inflammation could lead to the functional exhaustion of BM HPCs [19,22,28]. Our recent study indicated that depletion of pDCs efficiently rescued human CD45 cell reconstitution in humanized mice with chronic HIV-1 infection [29]. Thus, we hypothesized that pDCs may be responsible for the depletion of CD34^+^CD38^-^ early HPCs and their functional impairment during chronic HIV-1 infection.

**Fig 5:**
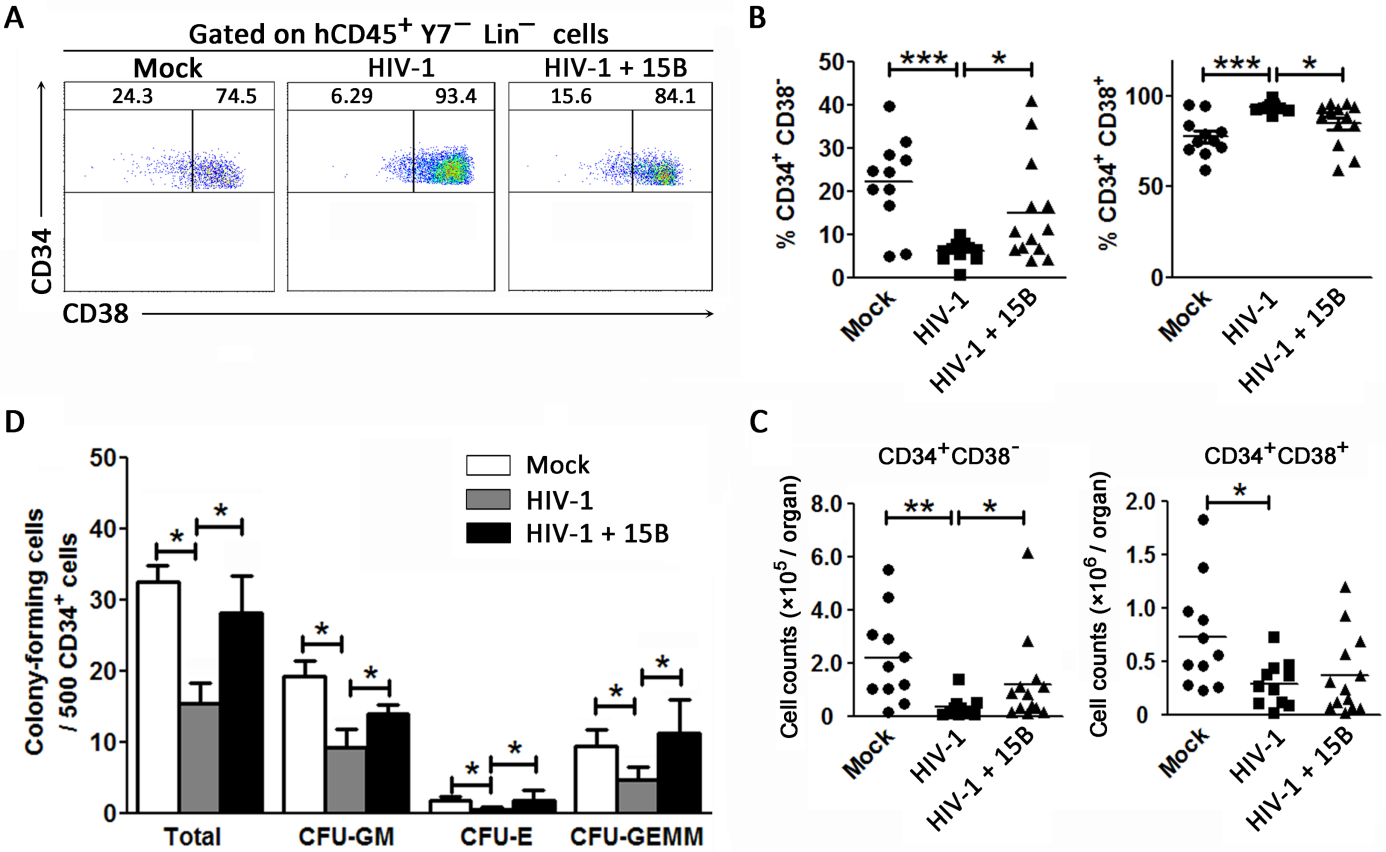
Depletion of pDCs during chronic HIV-1 infection rescues early CD34^+^CD38^-^ HPCs in humanized mice. (A) Representative dot plots showing the recovery of early CD34^+^CD38^-^ HPCs in BM of chronic HIV-1-infected humanized mice with pDC depletion. Numbers indicate percentages of early CD34^+^CD38^-^ HPCs and intermediate CD34^+^CD38^+^ HPCs in various groups of mice. (B and C) Summary data of percentages (B) and absolute cell numbers (C) of early CD34^+^CD38^-^ HPCs and intermediate CD34^+^CD38^+^ HPCs from BM in mock (n = 11), HIV-1-infected (n = 12) and HIV-1-infected humanized mice with pDC depletion (n = 13). Each dot represents one mouse. *P < 0.05 and ***P < 0.001 (two-tailed unpaired Student’s t-test). (D) Summary data of CFU that developed from CD34^+^ HPCs of mock-infected mice (n = 4), HIV-1-infected mice (n = 5) and HIV-1-infected mice with pDC depletion (n = 4). *P < 0.05 (two-tailed unpaired Student’s t-test). Error bars, s.e.m

To address the role of pDCs in the impairment of CD34^+^CD38^-^ HPCs in HIV-1 infection, humanized mice with chronic HIV-1 infection were treated with a pDC-depleting antibody (15B) as in our previous report [29]. Similarly, the plasma viral load was increased upon the depletion of pDCs and maintained at a higher level until termination (S4A Fig). Notably, the depletion of pDCs significantly changed the percentage of CD34^+^CD38^-^ early HPCs in the BM from humanized mice with chronic HIV-1 infection (Fig 5A). Pooled data further confirmed that both the percentages and cell counts of CD34^+^CD38^-^ early HPCs were restored by depletion of pDCs in HIV-1-infected mice (Fig 5B and 5C). In contrast, pDC depletion did not influence the percentages of CD34^+^CD38^-^ early HPCs (CD38 expression) and their proliferation *in vivo* indicated by BrdU expression in the BM in the absence of HIV-1 infection (S4B Fig). In addition, the cell counts of CD34^+^CD38^+^ intermediate HPCs showed only minor recovery by the depletion of pDCs during chronic HIV-1 infection, which is consistent with the slight decrease in proportion of CD34^+^CD38^+^ intermediate HPCs (Fig 5B and 5C).

Notably, only half of the animals showed a recovery in the proportion of CD34^+^CD38^-^ early HPCs after pDC depletion in Fig 5B, which prompted us to investigate whether the extent of pDC depletion affected the effectiveness of rescue of CD34^+^CD38^-^ early HPCs in humanized mice with chronic HIV-1 infection. We therefore divided the 13 animals into two groups; animals with less than the median percentage of CD34^+^CD38^-^ HPCs were placed in the "non-rescued" group (n = 6), while the others were included in the "rescued" group (n = 7). The rescued group was found to have significantly more CD34^+^CD38^-^ early HPCs and fewer CD34^+^CD38^+^ intermediate HPCs than the non-rescued group (S4C and S4D Fig). Importantly, the rescued mice showed a marked lack of pDCs in the BM and lower levels of IFN-α in plasma compared with non-rescued mice (S4E and S4F Fig). Accordingly, the rescued mice were also characterized by a higher level of HIV-1 replication than that of non-rescued mice (S4G Fig). Thus, we observed that the CD34^+^CD38^-^ early HPCs was negatively correlated with pDC percentages within CD45^+^ cells in BM of these HIV-1 infected humanized mice with pDC depletion (S4H Fig). These data indicated that the extent of rescue of CD34^+^CD38^-^ early HPCs was closely linked to *in vivo* pDC depletion rather than other potential causes such as HIV viral load in humanized mice with chronic HIV-1 infection.

To further qualify HPCs after pDC depletion, Lin^-^CD34^+^ cells were purified for colony-forming assays *ex vivo*. Cell colonies including GM, E and GEMM were found in culture (S3 Fig). The results demonstrated that pDC depletion could dramatically enhance CFU activity of the Lin^-^CD34^+^ cell population as well as increase the quantity of each colony type individually as compared with HIV-1-infected mice (Fig 5D).

### Depletion of pDCs restores gene expression profile of human CD34^+^ HPCs

**Fig 6:**
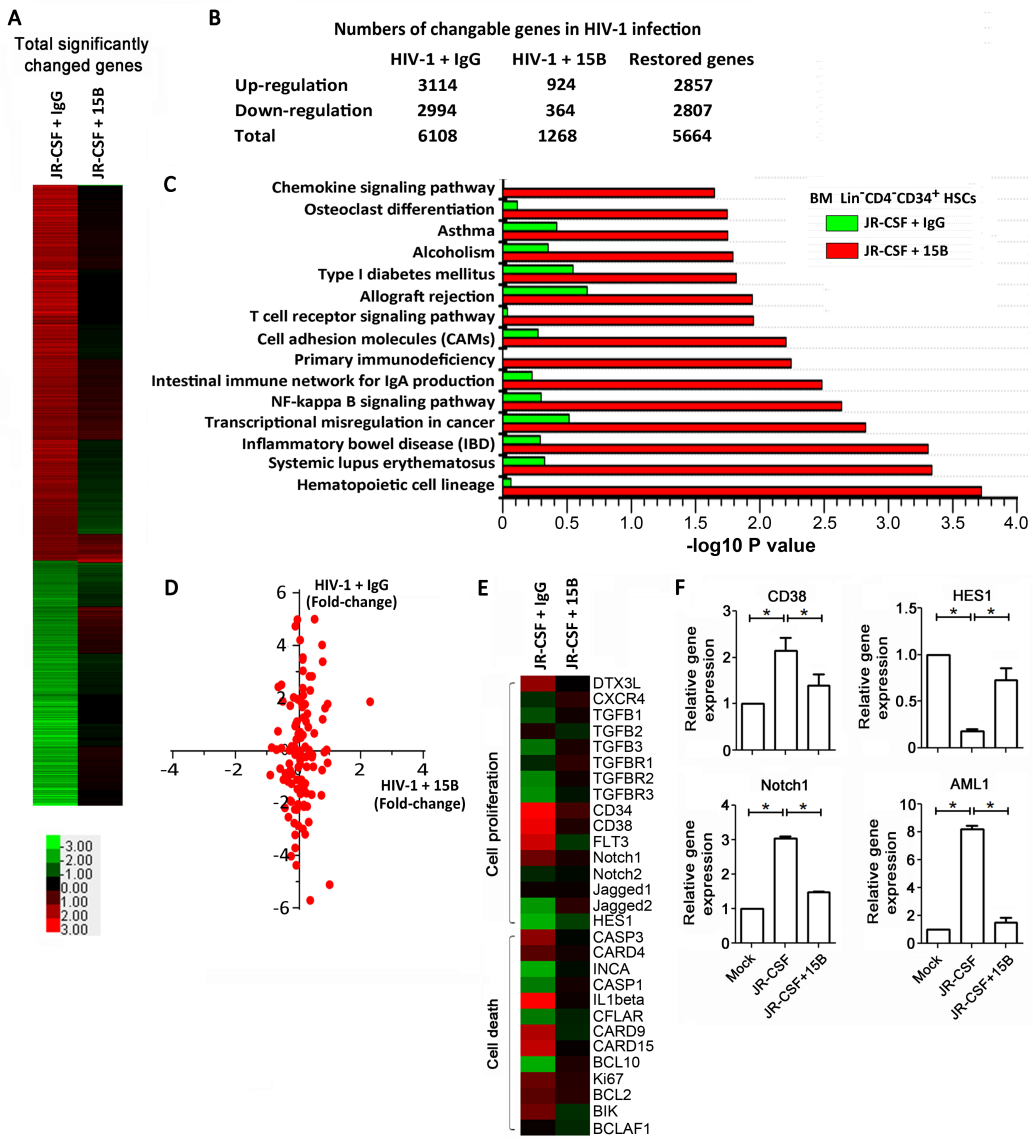
Depletion of pDCs reverses the dysregulated gene expression profile of human CD34^+^ HPCs affected by HIV-1 infection. Humanized mice were infected with HIV-1 JR-CSF and treated with 15B or control IgG at 11 weeks post-infection and terminated at 21 weeks post-infection. huCD45^+^Lin^-^CD34^+^ cells from BM of mock, HIV-1/IgG or HIV-1/15B treated mice were purified by flow cytometry (> 95% purity). Total mRNA were isolated and used for the cDNA microarray assay. (A) Heat map showing the relative expression of 6108 significantly up- or down-regulated genes indicated by the color bars in huCD45^+^Lin^-^CD34^+^ cells from HIV-1-infected over mock-infected samples (green, suppressed genes; red, induced genes. Fold change ≥ 2). (B) Table showing numbers of genes significantly up-regulated and down-regulated in humanized mice with HIV-1 infection treated with control IgG or 15B antibody for pDC depletion. (C) Pathway maps representing a set of signaling and metabolic maps. Sorting was performed for statistically significant maps. Experimental data are represented as green (JR-CSF + IgG) and red (JR-CSF + 15B) histograms. The height of the histogram corresponds to the relative expression of particular pathways. (D) Fold changes of 88 genes from hematopoietic cell lineage in HIV-1-infected mice treated with control IgG or 15B antibody for pDC depletion in relation to mock controls. (E) Expression levels of genes associated with HPC proliferation, differentiation and cell death in huCD45^+^Lin^-^CD34^+^ cells are indicated by the heat-map color bars (green, suppressed genes; red, induced genes. Fold change ≥ 2). (F) Summary data of the expression of HPC-associated genes, including CD38, HES1, Notch-1 and AML-1 in huCD45^+^Lin^-^CD34^+^ cells from various groups of mice (n = 4 per group). Data are shown as the mean ± s.e.m. *P < 0.05 (two-tailed unpaired Student’s t-test).

To understand how pDCs contribute to the impairment of HPCs during chronic HIV-1 infection, we analyzed gene expression of human HPCs in BM from HIV-1 chronically infected humanized mice. Human Lin^-^CD34^+^ cells from BM of humanized mice were isolated and submitted for gene expression analysis by cDNA array (Fig 6A), as described in a previous study [29]. A total of 3114 genes were significantly up-regulated, and 2994 genes were down-regulated spontaneously in CD34^+^ HPCs from HIV-1-infected humanized mice as compared to mock-treated mice (fold change > 2, Fig 6B and S5 Fig). Astonishingly, pDCs depletion during chronic HIV-1 infection in mice restored most of the interferon-stimulating genes (ISGs) to levels found in non-infected animals (S6A and S6B Fig). Along with the recovery of ISG gene expression, only 924 genes were significantly up-regulated, and 364 genes were down-regulated in BM CD34^+^ HPCs with pDCs depletion as compared to mock-treated mice (fold change > 2, Fig 6B and S5 Fig). Thus, pDCs depletion resulted in a restoration of a total of 5664 genes among 6108 genes (> 92.7%) that changed during HIV-1 infection in humanized mice, drawing back the whole gene expression profile to a pattern quite similar to that in mock-infected mice (Fig 6B and S5 Fig).

We then searched the GeneGo database to identify potentially relevant pathways in the genes influenced by chronic HIV-1 infection. The hematopoietic cell lineage was the most affected pathway induced by chronic HIV-1 infection among the top 15 pathways, whereas those dysregulated genes were also significantly restored to mock levels in pDC-depleted BM CD34^+^ HPCs of humanized mice chronically infected with HIV-1 (Fig 6C). We comprehensively analyzed the 88 genes in the hematopoietic cell lineage pathway (S7A Fig) and found that HIV-1 infection induced a significant up-regulation of 27 genes and down-regulation of 28 genes in humanized mice relative to mock controls (S7B Fig). However, depletion of pDCs during HIV-1 infection only induced a significant change in expression of one gene in relation to mock controls (S7B Fig). Summarized data further indicated that although HIV-1 infection led to a significant change of most of the genes with more than a 2-fold change, pDC depletion could attenuate the up-regulation of genes and restored the down-regulated genes to normal levels during chronic HIV-1 infection (Fig 6D and S7C Fig). In particular, some genes related to the HPC quiescent state (*DTX3L* and *CXCR4*) [30], colony forming capacity (*CD34*, *CD38*, *FLT3* and *TGFBR1-3*) [31], self-renewal and expansion capacity (*Notch1-2*, *Jagged1-2* and *Hes-1*) [32,33], as well as several important cell death genes were significantly altered by chronic HIV-1 infection, while pDCs depletion in HIV-1-infected humanized mice largely restored the abnormal expression of these genes to a similar pattern as seen in mock-infected mice (Fig 6E and 6F, S1 Table).

Simultaneously, we also performed mRNA expression analysis using spleen-derived CD45^+^ cells (S8A Fig). A total of 4757 genes were significantly up-regulated or down-regulated in splenic CD45^+^ cells from HIV-1-infected humanized mice as compared to mock-infected mice (fold change > 2, S8B Fig). The depletion of pDCs resulted in a restoration of about 44.0% of genes (2092/4757) of splenic cells changed by HIV-1 infection in humanized mice to a pattern quite similar to that in mock-infected mice (S8B Fig). The pathway analysis indicated that systemic lupus erythematosus (SLE) was the most affected pathway induced by chronic HIV-1 infection (S8C Fig), whereas the dysregulated genes expressed by splenic CD45^+^ cells were not significantly restored to mock levels in pDC-depleted humanized mice chronically infected with HIV-1 (S8D Fig). We also analyzed 127 genes in the SLE pathway changed by HIV-1 infection (S8E Fig) and found that HIV-1 infection induced significant changes of 48 genes relative to mock controls. However, depletion of pDCs during HIV-1 infection also induced significant changes in expression of 115 genes in relation to mock controls (S8E Fig), indicating that pDCs depletion failed to restore the changes in gene expression by spleen CD45^+^ cells to normal levels during chronic HIV-1 infection (S8F Fig).

These data strongly suggest that pDCs has relatively unique effects on the numerical reduction and functional impairment of BM CD34^+^ HPCs in HIV-1 chronically infected humanized mice, contributing to the suppression of hematopoiesis characterized by dysregulation of gene expression profiles.

We finally tested whether IFN-I directly up-regulates CD38 expression on HPCs *in vitro*. As shown in S9A and S9B Fig, neither IFN-α nor IFN-β showed any significant effect on CD38 expression on CD34^+^ cells *in vitro*. In addition, IFN-I culture did not affect CD34^+^ HPC expansion (S9C Fig). These data indicate that IFN-I did not directly affect CD38 expression and expansion of CD34^+^ HPC cells *in vitro*.

## Discussion

The present study demonstrates, for the first time, that human CD34^+^CD38^-^ early HSCs are subject to preferential depletion and functional impairment *in vivo* in the BM of humanized mice with chronic HIV-1 infection, in a pDC-dependent fashion. This study thus reveals a new target for the development of novel drugs targeting pDC activity to treat hematopoietic disorders during chronic HIV-1 infection and also demonstrates the utility of humanized mice to investigate important questions on HIV-1-mediated hematological abnormalities in the BM *in vivo*.

Previous studies have attempted to delineate the mechanism by which HIV-1 infection induces the impairment of HPCs, but it remains an intractable question due to the lack of a suitable experimental animal model that closely mimics human hematopoiesis during an ongoing HIV-1 infection *in vivo*. Here, we provide evidence that both early and intermediate HPCs are functionally developed and maintained for long-term self-renewal in the BM of humanized mice *in vivo*. This animal model has been demonstrated to be persistently infected by various strains of HIV-1 and develop major immune pathogenesis induced by acute or chronic HIV-1 infection as observed in HIV-1 patients [29,34–36]. Importantly, Nixon *et al*. made initial advances toward adopting a humanized mouse model to investigate an HIV-1 infection-induced hematopoiesis disorder [13]. Our present study underscores the rationale for utilizing a humanized mouse model to study the impact of HIV-1 infection on hematopoiesis *in vivo*. Taken together, these studies support the humanized mouse model as an experimentally amenable *in vivo* system for investigating HIV-1-associated pathogenesis.

Accumulating evidence has demonstrated that patients with long-term HIV-1 infection exhibit a deficiency in hematopoiesis [1,4,5], although the stage at which HPCs are impaired by HIV-1 infection is unclear. Nixon *et al*. showed that HPCs were susceptible to HIV-1 infection *in vitro* and *in vivo* in humanized mice and concluded that direct infection of intermediate CD34^+^CD38^+^ HPCs by HIV-1 adversely affected their hematopoietic potential and correlated with the observed pancytopenia in HIV-1 infected patients [13]. In this study, we found that CD34^+^CD38^-^ early HPCs were preferentially depleted during chronic HIV-1 infection, which correlated with the depression of hematopoiesis development and dysregulated gene expression in bulk Lin^-^CD34^+^ HPCs. However, our study failed to detect significant productive infection of Lin^-^CD34^+^ HPCs in the BM *in vivo* even with pDC depletion, as the depletion of pDCs led to dramatically increased viral replication. One possible explanation for this finding is that different HIV-1 strains may exhibit discrepancies during infection of HPCs *in vivo*. Future studies should examine the effect on HPC subsets by infection with other HIV-1 strains with different tropisms.

The mechanisms leading to abnormal hematopoiesis have not been clearly addressed in HIV-1 infection. Aside from the direct infection of HPC subsets, pDCs are possibly critical factors leading to hematopoietic suppression during HIV-1 infection, as the depletion of pDCs rescued early HPCs and their hematopoiesis. Available lines of evidence have also demonstrated that pDCs substantially mediate detrimental effects during chronic HIV-1 infection *in vivo*, even while they inhibit viral replication [29,37–39]. In addition, pDCs can secrete other pro-inflammatory cytokines, including TNF-α and IL-6. These chronic inflammatory cytokines can lead to exhaustion of hematopoiesis [19,22,28]. Most importantly, pDCs are the major IFN-I-producing cells during HIV-1 infection [29,40,41]. Currently, IFN-I is perhaps the primary contributor, since depletion of pDCs completely abolished IFN-I responses in humanized mice with chronic HIV-1 infection [29]. IFN-I has been recently demonstrated to be actively involved in immune pathogenesis of chronic virus infection [39,42–45]. Our data indicate that IFN-I did not directly affect CD38 expression on HPCs *in vitro* during short-term culture although it possibly promote the maturation of embryonic hematopoietic stem cells [46]. Other factors associated with chronic HIV-1 infection may contribute to IFN-induced HPC depletion and will be investigated in future study. Of course, we could not exclude the possibility that HIV-1 products are involved in the dysregulation of hematopoietic development. For example, HIV-1 Nef has been found to be responsible for hematopoietic defects of the BM in HIV-1 infection, dependent on the presence and activation of the PPARγ signaling pathway [17]. Thus, pDCs may contribute to abnormal hematopoiesis during chronic HIV-1 infection directly through viral infection or indirectly via diverse cytokines. Taking these studies into consideration, we also propose that HIV-1 infection may affect HPC function through multiple mechanisms (S10 Fig). Future studies should investigate in detail the individual factors responsible for compromising hematopoietic activity.

In summary, pDCs play a pivotal role in the immune-pathogenesis and hematopoiesis depression induced by chronic HIV-1 infection. This study, therefore, provides new insight into HIV-1-induced dysregulation of hematopoiesis and provides a novel strategy for treating abnormal hematopoiesis during chronic HIV-1 infection.

## Materials and methods

### Ethics statement

Approval for animal work was obtained from the University of North Carolina Institutional Animal Care and Use Committee (IACUC ID: 14–100). The study protocol on human samples was approved by the Institutional Review Board and the Ethics Committee of Beijing 302 Hospital in China. The written informed consent was obtained from each subject. Human BM samples were obtained from adult donors with liver transplantation as healthy controls and from HIV-1-infected adult patients for pathological diagnosis. Human fetal livers and thymuses (gestational age 16 to 20 weeks) were obtained from medically indicated or elective termination of pregnancies through a non-profit intermediary working with outpatient clinics (Advanced Bioscience Resources, Alameda, CA). Written informed consent from the maternal donor was obtained in all cases under regulations governing the clinic. All animal studies were conducted following NIH guidelines for housing and care of laboratory animals. The project was reviewed by the University’s Office of Human Research Ethics, which determined that this submission does not constitute human subjects research as defined under federal regulations [45 CFR 46.102 (d or f) and 21 CFR 56.102(c)(e)(l)].

### Construction of humanized mice

We constructed humanized Balb/c *rag2-γc* (DKO) mice and *Nod*-*rag1-γc* (NRG) mice (The Jackson Laboratory) in a similar manner as previously reported [36]. Briefly, human CD34^+^ cells were isolated from 16- to 20-week-old fetal liver tissues (Advanced Bioscience Resources, Alameda, CA). Tissues were digested with liver digest medium (Invitrogen, Frederick, MD). The suspension was filtered through a 70-μm cell strainer (BD Falcon, Lincoln Park, NJ) and centrifuged at 150 × *g* for 5 minutes to isolate mononuclear cells by Ficoll. After selection with the CD34^+^ magnetic-activated cell sorting (MACS) kit, CD34^+^ HPCs (0.5 × 10^6^) were injected into the liver of each 2- to 6-day-old DKO or NRG mice, which had been previously irradiated at 300 rad. More than 95% of the humanized mice were stably reconstituted with human leukocytes in the blood (60–90% at 12–14 weeks). Each cohort had similar levels of engraftment. All mice were housed at the University of North Carolina at Chapel Hill.

### HIV-1 virus stocks and infection of humanized mice

An R5-tropic strain of HIV-1, JR-CSF, was used for chronic HIV-1 infection. All viruses were generated by transfection of 293 T cells (SIGMA-ALORICH, Cat#12022001-1VL) with pYK-JRCSF (NIH AIDS reagents program, Cat# 2708). Humanized mice with stable human leukocyte reconstitution were infected with JR-CSF at a dose of 10 ng p24/mouse, through intravenous (i.v.) injection. Humanized mice infected with 293 T mock supernatant were used in control groups (S2 Fig). Viral genomic RNA in plasma was extracted using the QIAamp Viral RNA Mini Kit (QIAGEN, Cat# 52904) according to the manufacturer’s instruction. HIV-1 replication (genome copies/ml plasma) was measured by real-time PCR (ABI Applied Biosystem) or by p24-FACS detection of productively infected human T cells.

### Depletion of human pDCs in humanized mice

A monoclonal antibody specific to blood dendritic cell antigen-2 (BDCA2), 15B, was used to treat humanized mice through intra-peritoneal (i.p.) injection (4 mg/kg) as previously reported [29]. Briefly, 15B was applied to mice at 7 weeks post-infection by injecting twice every week for another 4 weeks.

### Animal termination and tissue processing

For chronic JR-CSF infection, mice were terminated at 12 week post-infection. On termination, total leukocytes were isolated from mouse lymphoid organs as previously described [29,34–36]. Lymphoid tissues, including peripheral blood (PB), peripheral lymph nodes (pLN), mesenteric lymph nodes (mLN), spleen and BM were harvested for analysis. Red blood cells were lysed with ACK buffer, and the remaining cells were stained and fixed with 1% (wt/vol) formaldehyde before FACS analysis. The total cell number was quantified by using Guava Easycytes with Guava Express software (Guava). Human BM cells were isolated by ficoll-hypaque density gradient centrifugation and collected for further analysis.

### Antibodies and flow cytometry

Surface and intracellular fluorochrome-conjugated antibodies or reagents from Biolegend, BD Bioscience, eBioscience and R&D Systems were used in this study. For humanized mice, live human leukocytes (Y7^-^mCD45^-^hCD45^+^) were analyzed for HPC subsets or phenotypic expression by using the CyAn FACS instrument (Dako). Live/dead fixable violet dead cell dye (LD7) was purchased from Molecular Probes (Eugene, OR). For intracellular p24 staining, freshly isolated cells were collected for surface staining, followed by cell permeabilization using a Cytofix/Cytoperm kit (BD Bioscience) and intracellular staining and washing. The data were analyzed using Summit Software.

### BrdU labeling *in vivo*

5-Bromo-2’-deoxyuridine (BrdU, Cat#: B5002, Sigma-Aldrich, St. Louis, MO) was first dissolved in water at a concentration of 10 mg/mL for stock in -20°C. The BrdU stock was then diluted in 200 μL PBS and injected i.p. at 100 mg/kg body weight. Four hours later, the mice were terminated, and BM cells were collected. BrdU staining was performed according to the manufacturer’s instructions. In brief, cells were first stained for surface markers and then incubated with a working solution of the BrdU staining buffer for 15 minutes, followed by incubation with DNase I (BIO-RAD, Cat#: 7326828) for 1 hour at 37°C in the dark. Thereafter, the cells were stained with the FITC-conjugated anti-BrdU antibody for 30 minutes at room temperature in the dark and subsequently washed. The data were analyzed using Summit Software.

### Colony-forming assays

The EasySep human CD34^+^ selection kit (Cat#:18056, StemCell Tech, Canada) was used to isolate CD34^+^ cells from frozen BM cells. The purity of CD34^+^ cells was greater than 90%. The CD34^+^ cells were then counted and seeded in complete methylcellulose (Methocult H04034; Stem Cell Technologies) at a concentration of 500 cells/mL and plated in 35-mm grid plates, 1 mL/plate, in triplicate per mouse according to the manufacturer’s instructions. Colonies were counted 2 weeks later in a blinded fashion using a QImaging Micropublisher 3.3 CCD digital camera and QCapture software version 3.0 (QImaging, Surrey, BC).

### Cell purification by FACS sorting

BM cells were pooled by mouse groups for human CD45^+^ cells sorting. Cells were stained with human CD45, mouse CD45 and 7-Aminoactinomycin D (7-AAD). For human CD34^+^ HSC sorting, anti-lineage (anti-CD3, anti-CD14, anti-CD16, anti-CD19, anti-CD20 and anti-CD56) and anti-CD34 antibodies were added to the antibody mix. Cell sorting was performed by the UNC Flow Cytometry Core.

### *In vitro* HPC maturation and expansion experiments

CD34^+^ cells were isolated from human fetal liver tissues. Then the cells were cultured in StemSpan SFEM medium (Stem Cell Technologies) with heparin (10 μg/ml, Sigma), recombinant human SCF (20 ng/ml, R&D), thrombopoietin (40 ng/ml, Cell Sciences) and CHIR99021 (GSK3 inhibitor, 250 nM, STEMGENT) for 48 hours in the presence of IFN-α or IFN-β at the dose of 20 IU/ml and 200 IU/ml, respectively. The cells were counted and collected for the detection of CD38 expression on HPCs.

### Agilent microarray assay

RNA purification was carried out using the RNeasy Plus Mini Kit (Cat# 74134, QIAGEN, Venlo, Limburg, Netherlands) according to the manufacturer’s instructions. DNase (QIAGEN) treatment was added to the column to eliminate any potential DNA contamination during RNA preparations. Total RNA was checked for quantity, purity and integrity by capillary electrophoresis. RNA was amplified with Cy3- and Cy5-labeled CTP in separate reactions to produce differentially labeled samples and reference cDNAs. Total RNA (200 ng to 400 ng) was used as the starting material to prepare cDNA. Both samples were hybridized to the same microarray (UNC Genomic and Bioinformatics Core) using SurePrint G3 Human Gene Expression 8 ◊ 60K Microarray Kit (Agilent). Agilent Feature Extraction v18 software was used to analyze all images. Gene expression values were quantified by the log2 ratio of the red channel intensity (mean) vs. green channel intensity (mean), followed by LOWESS normalization to remove the intensity-dependent dye bias.

### Statistical analysis

Data were analyzed using GraphPad Prism software version 5.0 (GraphPad software, San Diego, CA). Data from different cohorts of mice were compared using a 2-tailed unpaired T test. All results were considered significant for *P* values < 0.05.

## Supporting information

S1 Table The expression of HSC-associated genes in HIV-infected mice with or without pDCs depletion. (DOCX)

S1 Fig Gating strategy for CD34^+^ HPCs from BM in humanized mice. After gating on lymphocytes (FSC-SSC), singlets and live human CD45^+^ cells, the lineage^-^CD34^+^ cells that remained were identified as total HPCs. Based on CD38 expression, HPCs were further divided into CD38^-^ early and CD38^+^ intermediate HPC subpopulations. The lineage markers included CD3, CD14, CD16, CD19, CD20 and CD56.
(TIF)

S2 Fig Establishment of persistent HIV-1 infection in humanized mice (n = 9). (TIF)

S3 Fig Representative pictures showing morphology of colonies differentiated from CD34^+^ HPCs in BM of humanized mice. CFU-GM, colony-forming unit-granulocyte, macrophage. CFU-E, colony-forming unit-erythroid. CFU-GEMM, colony-forming unit-granulocyte, erythroid, macrophage, megakaryocyte.
(TIF)

S4 Fig Dependence of recovery of CD34^+^CD38^-^ early HPCs on pDC depletion status. (A) Plasma HIV-1 load at the termination of humanized mice with HIV-1 infection with or without pDC depletion. (B) Depletion of pDCs did not influence the CD38 expression and BrdU expression on HPCs in the BM from humanized mice (each group, n = 3) in the absence of HIV-1 infection. (C-G) Differences in CD34^+^CD38^-^ early HPC proportion (C), CD34^+^CD38^+^ intermediate HPC proportion (D), BM pDC percentage (E), plasma IFN-α level (F) and plasma HIV-1 load (G) between rescued (n = 7) and non-rescued (n = 6) groups in humanized mice with HIV-1 infection after pDC depletion. Mice with less than the median percentage of CD34^+^CD38^-^ HPCs were defined as the non-rescued group (n = 6), while others were defined as the rescued group (n = 7). (H) Correlation analysis between the percentage of CD34^+^CD38^-^ HPCs and the percentage of pDCs among CD45^+^ cells in BM of HIV-1 infected humanized mice with pDC depletion (Spearman correlation test). r, correlation coefficient; *P* values are shown.
(TIF)

S5 Fig Distribution of significantly up-regulated or down-regulated gene numbers in BM CD34^+^ HPC cells from HIV-1-infected mice with IgG and 15B relative to mock mice. (TIF)

S6 Fig Relative expression of ISGs in BM CD34^+^ HPC cells from HIV-1-infected mice treated with IgG and 15B over mock mice. (A) Heat map showing the relative expression of ISGs indicated by the color bars in huCD45^+^Lin^-^CD34^+^ cells from HIV-1-infected mice treated with IgG and 15B relative to mock samples (green, suppressed genes; red, induced genes. Fold change ≥ 2). (B) Table showing relative expression levels of ISGs in HIV-1-infected mice treated with IgG and 15B relative to mock mice.
(TIF)

S7 Fig Expression of HPC-associated genes in BM CD34^+^ HPC cells from HIV-infected mice with or without pDC depletion. (A) Heat map showing relative expression of 88 genes in the hematopoietic cell lineage pathway indicated by the color bars in huCD45^+^Lin^-^CD34^+^ cells from HIV-1-infected mice over mock samples (green, suppressed genes; red, induced genes. Fold change ≥ 2). (B) Table showing numbers of significantly up-regulated or down-regulated genes in the HPC pathway in HIV-1-infected mice treated with IgG and 15B relative to mock mice. Δ change, D-value between HIV-1+IgG and HIV-1+15B. (C) Fold changes in expression of 88 genes from hematopoietic cell lineage in HIV-1-infected mice with or without pDC depletion in relation to mock mice.
(TIF)

S8 Fig Depletion of pDCs slightly changes the dysregulated gene expression profile of splenic CD45^+^ cells affected by HIV-1 infection. (A) Heat map showing the relative gene expression indicated by the colored bars in huCD45^+^ cells from the spleen of HIV-1-infected humanized mice over mock samples (green, suppressed genes; red, induced genes). (B) Table showing the number of genes significantly up-regulated and down-regulated in splenic CD45^+^ cells from HIV-1-infected humanized mice with or without pDC depletion. (C) Table showing the KEGG pathway analysis of the top 8 most altered pathways in splenic CD45^+^ cells from HIV-1-infected humanized mice relative to mock samples. (D) Heat map showing the relative gene expression of the SLE pathway indicated by the colored bars in splenic huCD45^+^ cells of HIV-1-infected humanized mice over mock samples (green, suppressed genes; red, induced genes). (E) Table showing numbers of significantly up-regulated or down-regulated genes in SLE pathway in HIV-1-infected mice with IgG and 15B relative to mock mice. Δ change, D-value between HIV-1+IgG minus HIV-1+15B. (F) Fold changes of 88 genes from SLE pathway in HIV-1-infected mice with or without pDC depletion in relation to mock mice.
(TIF)

S9 Fig IFN-I slightly up-regulated CD38 expression on HPCs *in vitro*. (A and B) Representative dot plots (A) and pool data (n = 3, B) indicated CD38 expression on purified CD34^+^ fetal liver-derived HPCs in vitro of 24- and 48-hours in the presence of IFN-α and IFN-β at 20 IU/ml and 200 IU/ml doses. The numbers in (A) indicated that the CD38 percentages on HPCs. * *p* < 0.05. (C) Pool data indicated the cell counts of CD34+ HPCs in vitro after 24- and 48-hour culture in the presence of IFN-I (n = 3).
(TIF)

S10 Fig A model of HIV-1-induced hematopoietic suppression via pDC-dependent mechanisms. HIV-1 infection activates pDCs, possibly through type I IFNs, IL-6 and TNF-α, suppresses self-renewal proliferation of early CD34^+^CD38^-^ HPCs and dysregulates gene expression profile in HPCs leading to their depletion and functional impairment. This effect on early HSCs subsequently contributes to HIV-1-induced pathogenesis such as loss of all human leukocyte cells (pancytopenia). Suppression of HIV-1 replication by ① cART, ② depletion of pDC or ③ blocking reagents against pDC-derived cytokines will restore HSC number and function.
(TIF)

## References

[1] Moses A, Nelson J, Bagby GC Jr. (1998) The influence of human immunodeficiency virus-1 on hematopoiesis. Blood 91: 1479–1495. 9473211

[2] Zon LI, Arkin C, Groopman JE (1987) Haematologic manifestations of the human immune deficiency virus (HIV). Br J Haematol 66: 251–256. 3606961 10.1111/j.1365-2141.1987.tb01307.x

[3] Burstein Y, Rashbaum WK, Hatch WC, Calvelli T, Golodner M, et al (1992) Alterations in human fetal hematopoiesis are associated with maternal HIV infection. Pediatr Res 32: 155–159. doi: 10.1203/00006450-199208000-00006 1508604 10.1203/00006450-199208000-00006

[4] Donahue RE, Johnson MM, Zon LI, Clark SC, Groopman JE (1987) Suppression of in vitro haematopoiesis following human immunodeficiency virus infection. Nature 326: 200–203. doi: 10.1038/326200a0 2434864 10.1038/326200a0

[5] Treacy M, Lai L, Costello C, Clark A (1987) Peripheral blood and bone marrow abnormalities in patients with HIV related disease. Br J Haematol 65: 289–294. 3567082 10.1111/j.1365-2141.1987.tb06855.x

[6] Sloand E (2005) Hematologic complications of HIV infection. AIDS Rev 7: 187–196. 16425959

[7] Marandin A, Katz A, Oksenhendler E, Tulliez M, Picard F, et al (1996) Loss of primitive hematopoietic progenitors in patients with human immunodeficiency virus infection. Blood 88: 4568–4578. 8977248

[8] Sloand EM, Young NS, Sato T, Kumar P, Kim S, et al (1997) Secondary colony formation after long-term bone marrow culture using peripheral blood and bone marrow of HIV-infected patients. AIDS 11: 1547–1553. 9365758 10.1097/00002030-199713000-00002

[9] Huang SS, Barbour JD, Deeks SG, Huang JS, Grant RM, et al (2000) Reversal of human immunodeficiency virus type 1-associated hematosuppression by effective antiretroviral therapy. Clin Infect Dis 30: 504–510. doi: 10.1086/313714 10722435 10.1086/313714

[10] Thiebot H, Vaslin B, Derdouch S, Bertho JM, Mouthon F, et al (2005) Impact of bone marrow hematopoiesis failure on T-cell generation during pathogenic simian immunodeficiency virus infection in macaques. Blood 105: 2403–2409. doi: 10.1182/blood-2004-01-0025 15388577 10.1182/blood-2004-01-0025

[11] Mercer EM, Lin YC, Murre C (2011) Factors and networks that underpin early hematopoiesis. Semin Immunol 23: 317–325. doi: 10.1016/j.smim.2011.08.004 21930392 10.1016/j.smim.2011.08.004 PMC3217090

[12] Novershtern N, Subramanian A, Lawton LN, Mak RH, Haining WN, et al (2011) Densely interconnected transcriptional circuits control cell states in human hematopoiesis. Cell 144: 296–309. doi: 10.1016/j.cell.2011.01.004 21241896 10.1016/j.cell.2011.01.004 PMC3049864

[13] Nixon CC, Vatakis DN, Reichelderfer SN, Dixit D, Kim SG, et al (2013) HIV-1 infection of hematopoietic progenitor cells in vivo in humanized mice. Blood 122: 2195–2204. doi: 10.1182/blood-2013-04-496950 23886835 10.1182/blood-2013-04-496950 PMC3785119

[14] Durand CM, Ghiaur G, Siliciano JD, Rabi SA, Eisele EE, et al (2012) HIV-1 DNA is detected in bone marrow populations containing CD4+ T cells but is not found in purified CD34+ hematopoietic progenitor cells in most patients on antiretroviral therapy. J Infect Dis 205: 1014–1018. doi: 10.1093/infdis/jir884 22275402 10.1093/infdis/jir884 PMC3282572

[15] McNamara LA, Onafuwa-Nuga A, Sebastian NT, Riddell Jt, Bixby D, et al (2013) CD133+ hematopoietic progenitor cells harbor HIV genomes in a subset of optimally treated people with long-term viral suppression. J Infect Dis 207: 1807–1816. doi: 10.1093/infdis/jit118 23554378 10.1093/infdis/jit118 PMC3654754

[16] Pace M, O'Doherty U (2013) Hematopoietic stem cells and HIV infection. J Infect Dis 207: 1790–1792. doi: 10.1093/infdis/jit120 23554379 10.1093/infdis/jit120 PMC3654756

[17] Prost S, Le Dantec M, Auge S, Le Grand R, Derdouch S, et al (2008) Human and simian immunodeficiency viruses deregulate early hematopoiesis through a Nef/PPARgamma/STAT5 signaling pathway in macaques. J Clin Invest 118: 1765–1775. doi: 10.1172/JCI33037 18431514 10.1172/JCI33037 PMC2323187

[18] Akkina R (2013) New insights into HIV impact on hematopoiesis. Blood 122: 2144–2146. doi: 10.1182/blood-2013-08-518274 24072846 10.1182/blood-2013-08-518274

[19] Essers MA, Offner S, Blanco-Bose WE, Waibler Z, Kalinke U, et al (2009) IFNalpha activates dormant haematopoietic stem cells in vivo. Nature 458: 904–908. doi: 10.1038/nature07815 19212321 10.1038/nature07815

[20] Baldridge MT, King KY, Boles NC, Weksberg DC, Goodell MA (2010) Quiescent haematopoietic stem cells are activated by IFN-gamma in response to chronic infection. Nature 465: 793–797. doi: 10.1038/nature09135 20535209 10.1038/nature09135 PMC2935898

[21] MacNamara KC, Jones M, Martin O, Winslow GM (2011) Transient activation of hematopoietic stem and progenitor cells by IFNgamma during acute bacterial infection. PLoS One 6: e28669 doi: 10.1371/journal.pone.0028669 22194881 10.1371/journal.pone.0028669 PMC3237486

[22] Sato T, Onai N, Yoshihara H, Arai F, Suda T, et al (2009) Interferon regulatory factor-2 protects quiescent hematopoietic stem cells from type I interferon-dependent exhaustion. Nat Med 15: 696–700. doi: 10.1038/nm.1973 19483695 10.1038/nm.1973

[23] Yang L, Dybedal I, Bryder D, Nilsson L, Sitnicka E, et al (2005) IFN-gamma negatively modulates self-renewal of repopulating human hemopoietic stem cells. J Immunol 174: 752–757. 15634895 10.4049/jimmunol.174.2.752

[24] Dybedal I, Bryder D, Fossum A, Rusten LS, Jacobsen SE (2001) Tumor necrosis factor (TNF)-mediated activation of the p55 TNF receptor negatively regulates maintenance of cycling reconstituting human hematopoietic stem cells. Blood 98: 1782–1791. 11535512 10.1182/blood.v98.6.1782

[25] Pronk CJ, Veiby OP, Bryder D, Jacobsen SE (2011) Tumor necrosis factor restricts hematopoietic stem cell activity in mice: involvement of two distinct receptors. J Exp Med 208: 1563–1570. doi: 10.1084/jem.20110752 21768269 10.1084/jem.20110752 PMC3149225

[26] Zhang Y, Harada A, Bluethmann H, Wang JB, Nakao S, et al (1995) Tumor necrosis factor (TNF) is a physiologic regulator of hematopoietic progenitor cells: increase of early hematopoietic progenitor cells in TNF receptor p55-deficient mice in vivo and potent inhibition of progenitor cell proliferation by TNF alpha in vitro. Blood 86: 2930–2937. 7579385

[27] Esplin BL, Shimazu T, Welner RS, Garrett KP, Nie L, et al (2011) Chronic exposure to a TLR ligand injures hematopoietic stem cells. J Immunol 186: 5367–5375. doi: 10.4049/jimmunol.1003438 21441445 10.4049/jimmunol.1003438 PMC3086167

[28] Zhao Y, Ling F, Wang HC, Sun XH (2013) Chronic TLR signaling impairs the long-term repopulating potential of hematopoietic stem cells of wild type but not Id1 deficient mice. PLoS One 8: e55552 doi: 10.1371/journal.pone.0055552 23383338 10.1371/journal.pone.0055552 PMC3562238

[29] Li G, Cheng M, Nunoya J, Cheng L, Guo H, et al (2014) Plasmacytoid dendritic cells suppress HIV-1 replication but contribute to HIV-1 induced immunopathogenesis in humanized mice. PLoS Pathog 10: e1004291 doi: 10.1371/journal.ppat.1004291 25077616 10.1371/journal.ppat.1004291 PMC4117636

[30] Tzeng YS, Li H, Kang YL, Chen WC, Cheng WC, et al (2011) Loss of Cxcl12/Sdf-1 in adult mice decreases the quiescent state of hematopoietic stem/progenitor cells and alters the pattern of hematopoietic regeneration after myelosuppression. Blood 117: 429–439. doi: 10.1182/blood-2010-01-266833 20833981 10.1182/blood-2010-01-266833

[31] Cardoso AA, Li ML, Batard P, Hatzfeld A, Brown EL, et al (1993) Release from quiescence of CD34+ CD38- human umbilical cord blood cells reveals their potentiality to engraft adults. Proc Natl Acad Sci U S A 90: 8707–8711. 7690969 10.1073/pnas.90.18.8707 PMC47427

[32] Butler JM, Nolan DJ, Vertes EL, Varnum-Finney B, Kobayashi H, et al (2010) Endothelial cells are essential for the self-renewal and repopulation of Notch-dependent hematopoietic stem cells. Cell Stem Cell 6: 251–264. doi: 10.1016/j.stem.2010.02.001 20207228 10.1016/j.stem.2010.02.001 PMC2866527

[33] Kunisato A, Chiba S, Nakagami-Yamaguchi E, Kumano K, Saito T, et al (2003) HES-1 preserves purified hematopoietic stem cells ex vivo and accumulates side population cells in vivo. Blood 101: 1777–1783. doi: 10.1182/blood-2002-07-2051 12406868 10.1182/blood-2002-07-2051

[34] Jiang Q, Zhang L, Wang R, Jeffrey J, Washburn ML, et al (2008) FoxP3+CD4+ regulatory T cells play an important role in acute HIV-1 infection in humanized Rag2-/-gammaC-/- mice in vivo. Blood 112: 2858–2868. doi: 10.1182/blood-2008-03-145946 18544681 10.1182/blood-2008-03-145946 PMC2556621

[35] Zhang L, Jiang Q, Li G, Jeffrey J, Kovalev GI, et al (2011) Efficient infection, activation, and impairment of pDCs in the BM and peripheral lymphoid organs during early HIV-1 infection in humanized rag2(-)/(-)gamma C(-)/(-) mice in vivo. Blood 117: 6184–6192. doi: 10.1182/blood-2011-01-331173 21505190 10.1182/blood-2011-01-331173 PMC3122941

[36] Zhang L, Kovalev GI, Su L (2007) HIV-1 infection and pathogenesis in a novel humanized mouse model. Blood 109: 2978–2981. doi: 10.1182/blood-2006-07-033159 17132723 10.1182/blood-2006-07-033159 PMC1852218

[37] Zhang Z, Cheng L, Zhao J, Li G, Zhang L, et al (2015) Plasmacytoid dendritic cells promote HIV-1-induced group 3 innate lymphoid cell depletion. J Clin Invest 125: 3692–3703. doi: 10.1172/JCI82124 26301812 10.1172/JCI82124 PMC4588300

[38] Cheng L, Ma J, Li J, Li D, Li G, et al (2017) Blocking type I interferon signaling enhances T cell recovery and reduces HIV-1 reservoirs. J Clin Invest 127: 269–279. doi: 10.1172/JCI90745 27941247 10.1172/JCI90745 PMC5199717

[39] Zhen A, Rezek V, Youn C, Lam B, Chang N, et al (2017) Targeting type I interferon-mediated activation restores immune function in chronic HIV infection. J Clin Invest 127: 260–268. doi: 10.1172/JCI89488 27941243 10.1172/JCI89488 PMC5199686

[40] O'Brien M, Manches O, Wilen C, Gopal R, Huq R, et al (2016) CD4 Receptor is a Key Determinant of Divergent HIV-1 Sensing by Plasmacytoid Dendritic Cells. PLoS Pathog 12: e1005553 doi: 10.1371/journal.ppat.1005553 27082754 10.1371/journal.ppat.1005553 PMC4833349

[41] Beignon AS, McKenna K, Skoberne M, Manches O, DaSilva I, et al (2005) Endocytosis of HIV-1 activates plasmacytoid dendritic cells via Toll-like receptor-viral RNA interactions. J Clin Invest 115: 3265–3275. doi: 10.1172/JCI26032 16224540 10.1172/JCI26032 PMC1253628

[42] Papatriantafyllou M (2013) Infection: the interferon paradox. Nat Rev Immunol 13: 392. 10.1038/nri3461 23648970

[43] Sandler NG, Bosinger SE, Estes JD, Zhu RT, Tharp GK, et al (2014) Type I interferon responses in rhesus macaques prevent SIV infection and slow disease progression. Nature 511: 601–605. doi: 10.1038/nature13554 25043006 10.1038/nature13554 PMC4418221

[44] Teijaro JR, Ng C, Lee AM, Sullivan BM, Sheehan KC, et al (2013) Persistent LCMV infection is controlled by blockade of type I interferon signaling. Science 340: 207–211. doi: 10.1126/science.1235214 23580529 10.1126/science.1235214 PMC3640797

[45] Wilson EB, Yamada DH, Elsaesser H, Herskovitz J, Deng J, et al (2013) Blockade of chronic type I interferon signaling to control persistent LCMV infection. Science 340: 202–207. doi: 10.1126/science.1235208 23580528 10.1126/science.1235208 PMC3704950

[46] Kim PG, Canver MC, Rhee C, Ross SJ, Harriss JV, et al (2016) Interferon-α signaling promotes embryonic HSC maturation. Blood 128: 204–216. doi: 10.1182/blood-2016-01-689281 27095787 10.1182/blood-2016-01-689281 PMC4946201

