# Supplementary material for: HIV-1 infection depletes human CD34+CD38- hematopoietic progenitor cells via pDC-dependent mechanisms
Source: PLoS Pathog. 2017 Jul 31;13(7):e1006505. doi: 10.1371/journal.ppat.1006505 (PMC5552321; doi:10.1371/journal.ppat.1006505)
Supplement: S1 Table — (DOCX) [file ppat.1006505.s001.docx]

**S1 Table**

**The expression of HSC-associated genes in HIV-infected mice with or without pDCs depletion**

| **Gene name** | **JR-CSF+IgG** | **JR-CSF+15B** |
| --- | --- | --- |
| **DTX3L** | **1.731** | **0.026** |
| **CXCR4** | **-0.53** | **0.5** |
| **TGFB1** | **-0.92** | **0.21** |
| **TGFB2** | **0.36** | **-0.44** |
| **TGFB3** | **-1.34** | **0.33** |
| **TGFBR1** | **-0.46** | **0.54** |
| **TGFBR2** | **-1.56** | **0.21** |
| **TGFBR3** | **-1.67** | **-0.23** |
| **CD34** | **3.37** | **0.79** |
| **CD38** | **2.81** | **0.37** |
| **FLT3** | **2.43** | **-0.63** |
| **Notch1** | **1.29** | **0.3** |
| **Notch2** | **-0.447** | **-0.109** |
| **Jagged1** | **0.16** | **0.12** |
| **Jagged2** | **-1.79** | **0.51** |
| **HES1** | **-2.08** | **-0.75** |
| **CASP3** | **1.662** | **-0.06** |
| **CARD4** | **1.02** | **0.23** |
| **INCA** | **-2.028** | **-0.147** |
| **CASP1** | **-1.45** | **0.25** |
| **IL1 beta** | **3.46** | **0.15** |
| **CFLAR** | **-1.43** | **-0.39** |
| **CARD9** | **2.083** | **-0.425** |
| **CARD15** | **2.29** | **0.08** |
| **BCL10** | **-2.033** | **0.375** |
| **Ki67** | **1.25** | **0.458** |
| **BCL2** | **1.44** | **0.08** |
| **BIK** | **1.333** | **-0.537** |
| **BCLAF1** | **0.111** | **-0.471** |

**The values are shown as relative gene expression over mock mice.**
