# Supplementary figures and images for: HIV-1 infection depletes human CD34+CD38- hematopoietic progenitor cells via pDC-dependent mechanisms
Source: PLoS Pathog. 2017 Jul 31;13(7):e1006505. doi: 10.1371/journal.ppat.1006505 (PMC5552321; doi:10.1371/journal.ppat.1006505)

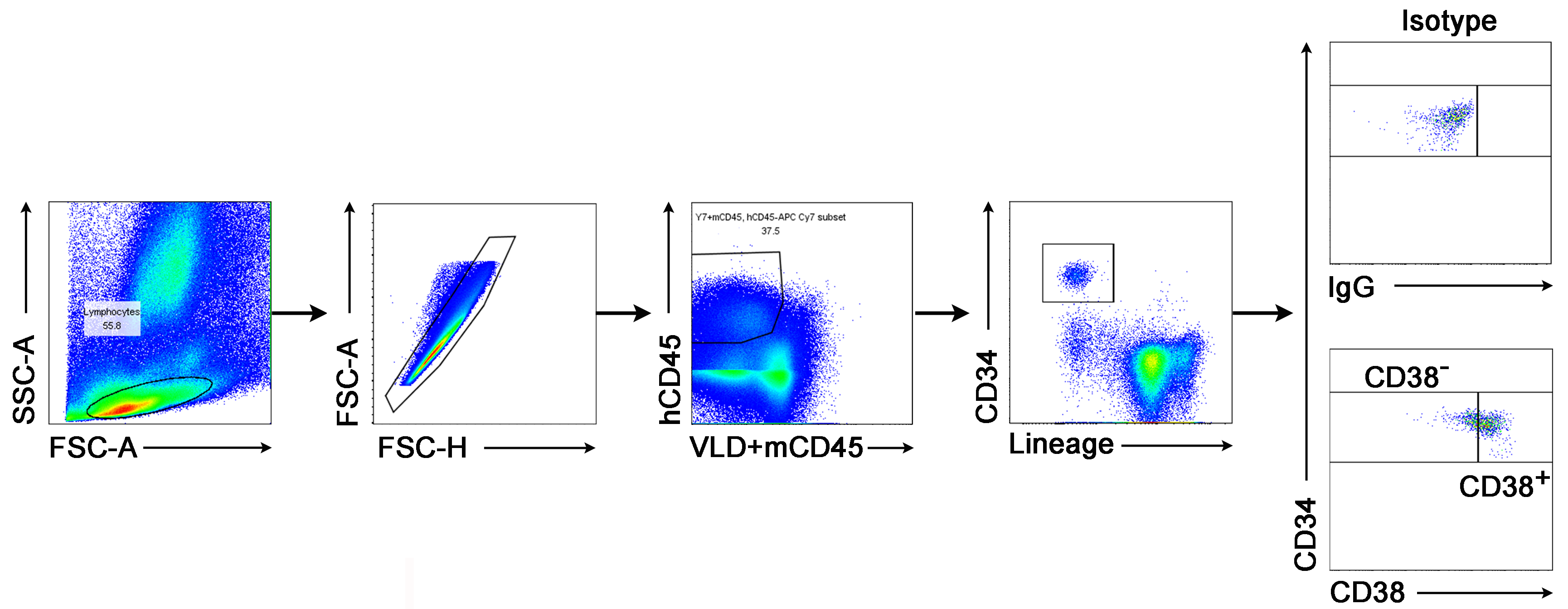

Supplement: S1 Fig — After gating on lymphocytes (FSC-SSC), singlets and live human CD45+ cells, the lineage-CD34+ cells that remained were identified as total HPCs. Based on CD38 expression, HPCs were further divided into CD38- early and CD38+ intermediate HPC subpopulations. The lineage markers included CD3, CD14, CD16, CD19, CD20 and CD56. (TIF) [file ppat.1006505.s002.tif]

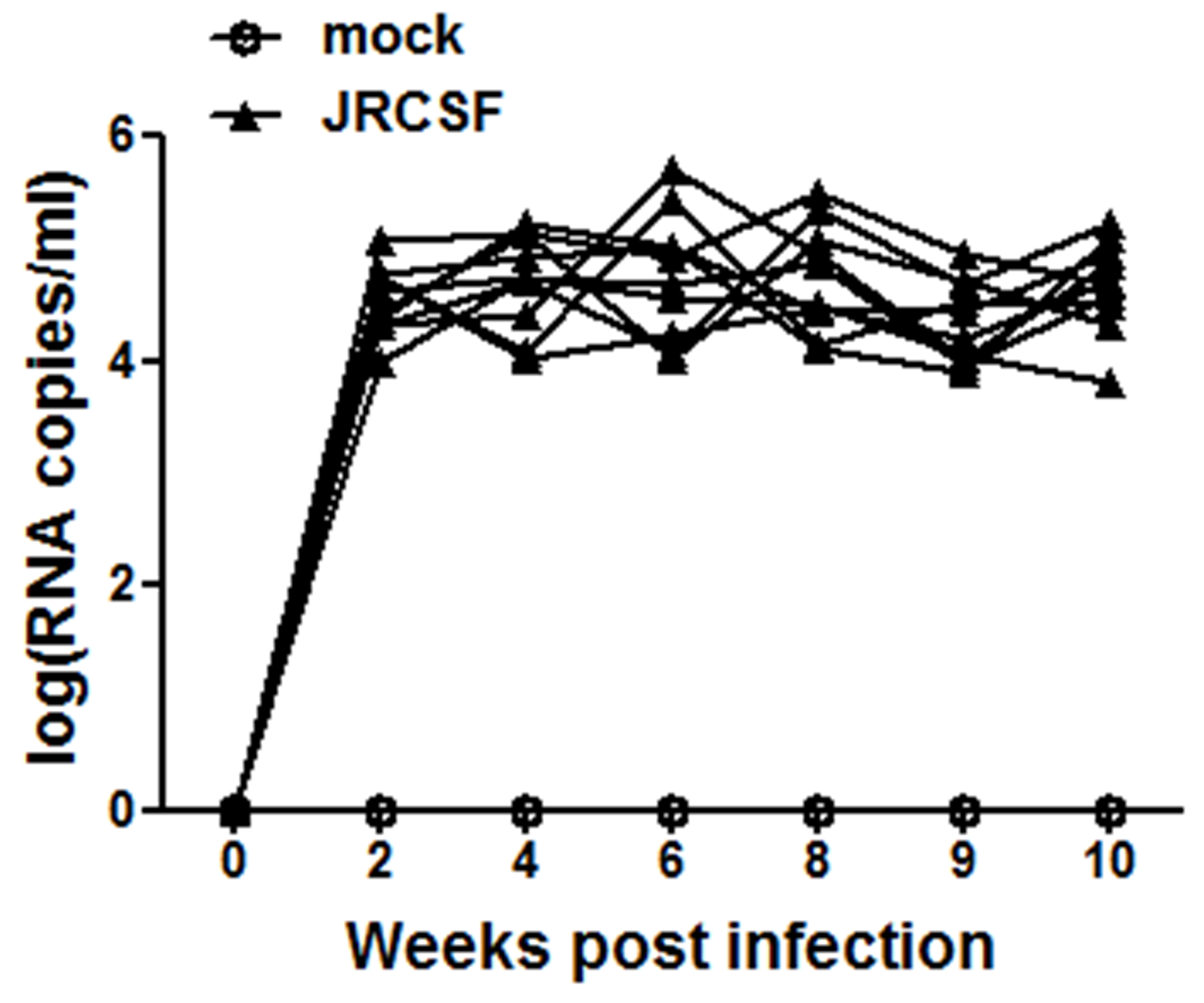

Supplement: S2 Fig — (TIF) [file ppat.1006505.s003.tif]

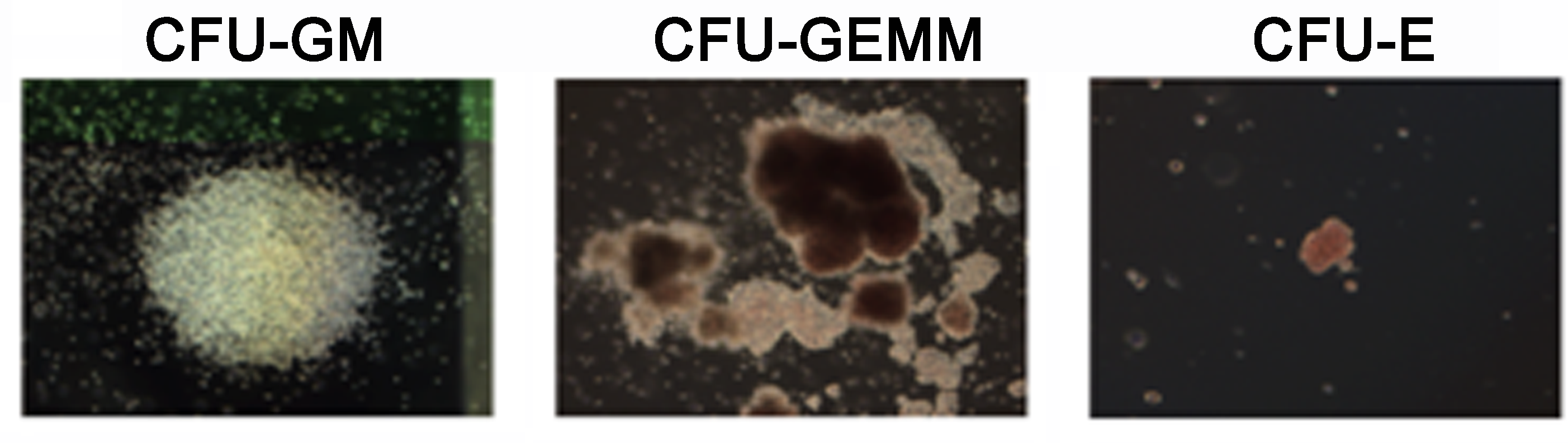

Supplement: S3 Fig — CFU-GM, colony-forming unit-granulocyte, macrophage. CFU-E, colony-forming unit-erythroid. CFU-GEMM, colony-forming unit-granulocyte, erythroid, macrophage, megakaryocyte. (TIF) [file ppat.1006505.s004.tif]

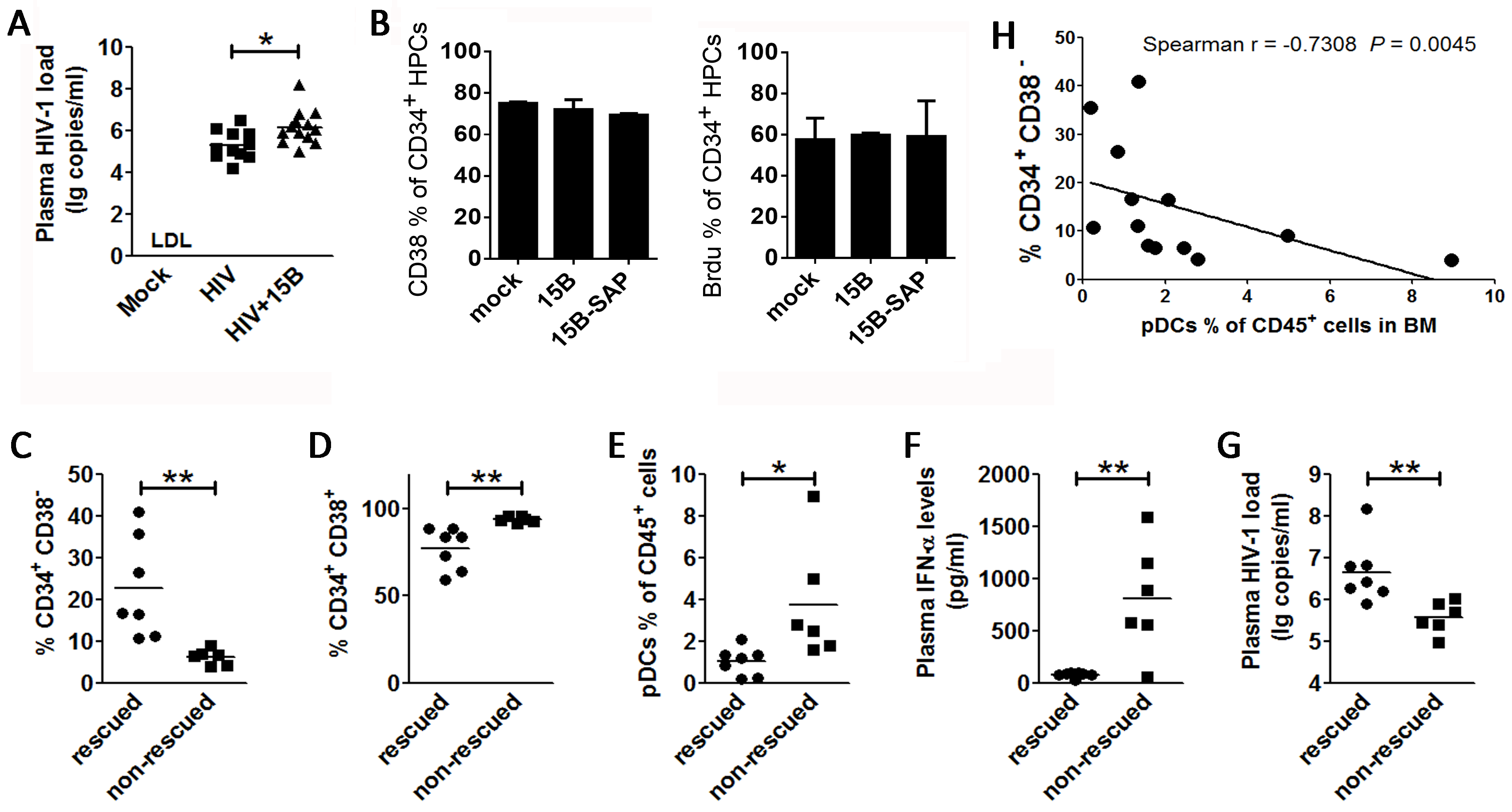

Supplement: S4 Fig — (A) Plasma HIV-1 load at the termination of humanized mice with HIV-1 infection with or without pDC depletion. (B) Depletion of pDCs did not influence the CD38 expression and BrdU expression on HPCs in the BM from humanized mice (each group, n = 3) in the absence of HIV-1 infection. (C-G) Differences in CD34+CD38- early HPC proportion (C), CD34+CD38+ intermediate HPC proportion (D), BM pDC percentage (E), plasma IFN-α level (F) and plasma HIV-1 load (G) between rescued (n = 7) and non-rescued (n = 6) groups in humanized mice with HIV-1 infection after pDC depletion. Mice with less than the median percentage of CD34+CD38- HPCs were defined as the non-rescued group (n = 6), while others were defined as the rescued group (n = 7). (H) Correlation analysis between the percentage of CD34+CD38- HPCs and the percentage of pDCs among CD45+ cells in BM of HIV-1 infected humanized mice with pDC depletion (Spearman correlation test). r, correlation coefficient; P values are shown. (TIF) [file ppat.1006505.s005.tif]

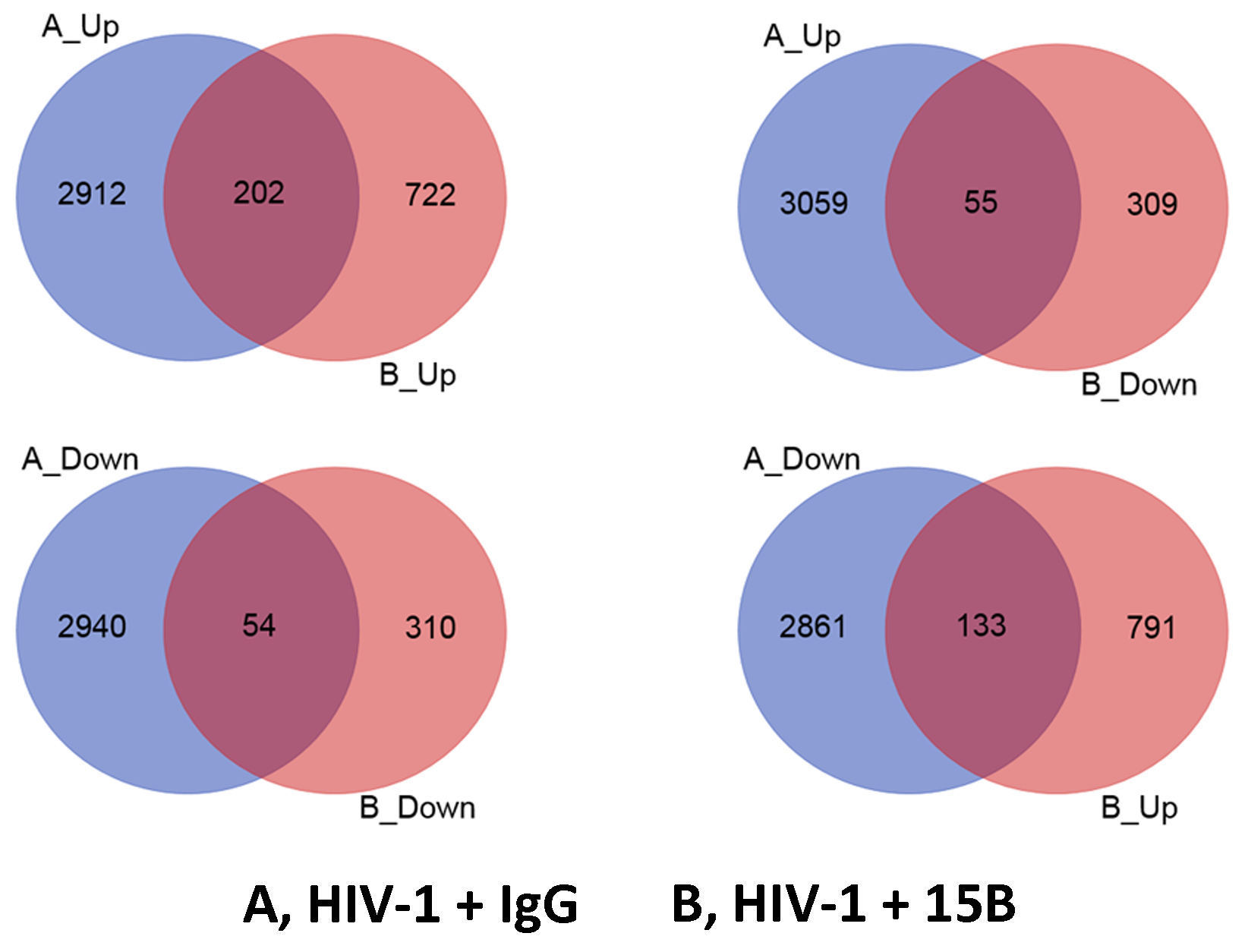

Supplement: S5 Fig — (TIF) [file ppat.1006505.s006.tif]

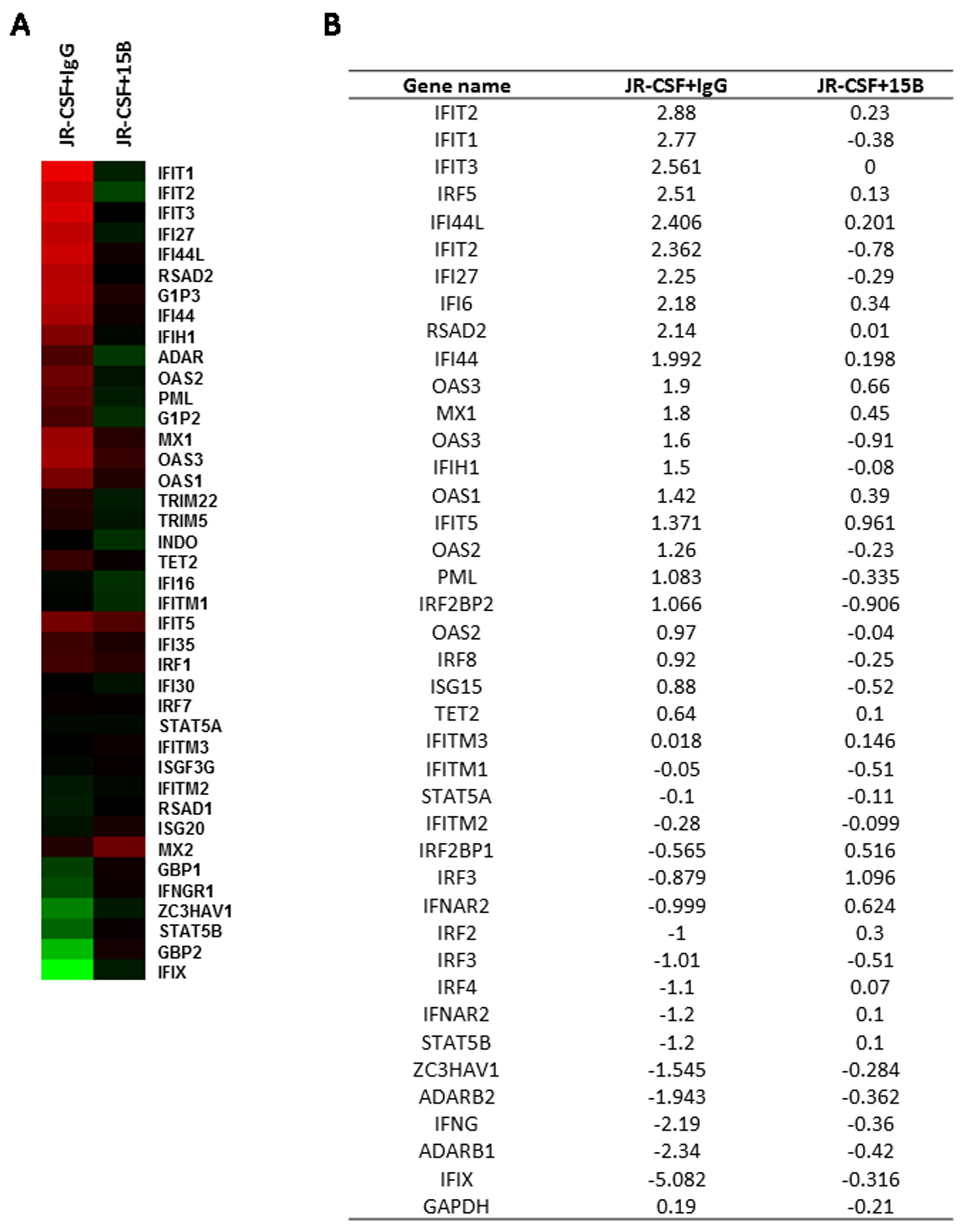

Supplement: S6 Fig — (A) Heat map showing the relative expression of ISGs indicated by the color bars in huCD45+Lin-CD34+ cells from HIV-1-infected mice treated with IgG and 15B relative to mock samples (green, suppressed genes; red, induced genes. Fold change ≥ 2). (B) Table showing relative expression levels of ISGs in HIV-1-infected mice treated with IgG and 15B relative to mock mice. (TIF) [file ppat.1006505.s007.tif]

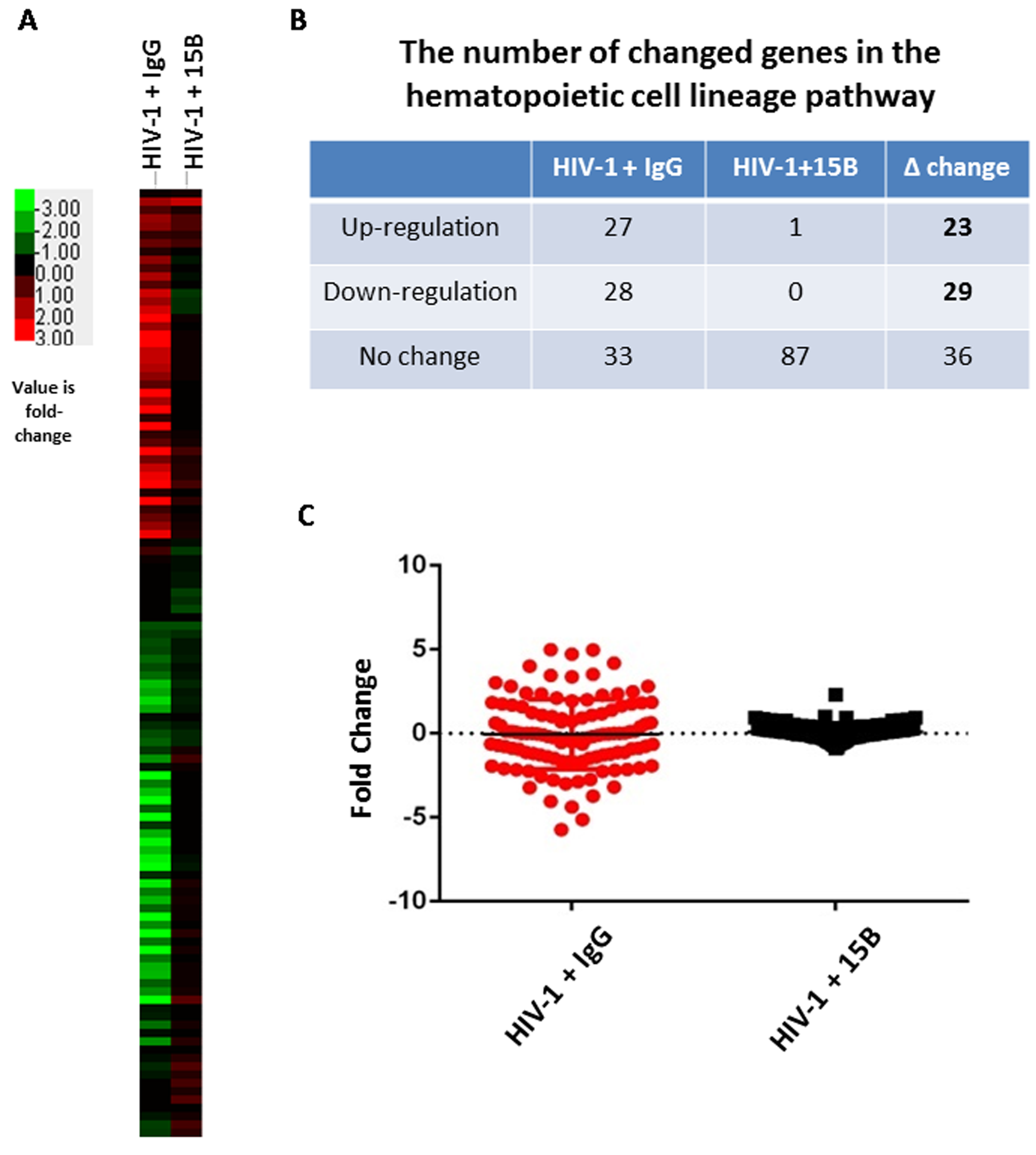

Supplement: S7 Fig — (A) Heat map showing relative expression of 88 genes in the hematopoietic cell lineage pathway indicated by the color bars in huCD45+Lin-CD34+ cells from HIV-1-infected mice over mock samples (green, suppressed genes; red, induced genes. Fold change ≥ 2). (B) Table showing numbers of significantly up-regulated or down-regulated genes in the HPC pathway in HIV-1-infected mice treated with IgG and 15B relative to mock mice. Δ change, D-value between HIV-1+IgG and HIV-1+15B. (C) Fold changes in expression of 88 genes from hematopoietic cell lineage in HIV-1-infected mice with or without pDC depletion in relation to mock mice. (TIF) [file ppat.1006505.s008.tif]

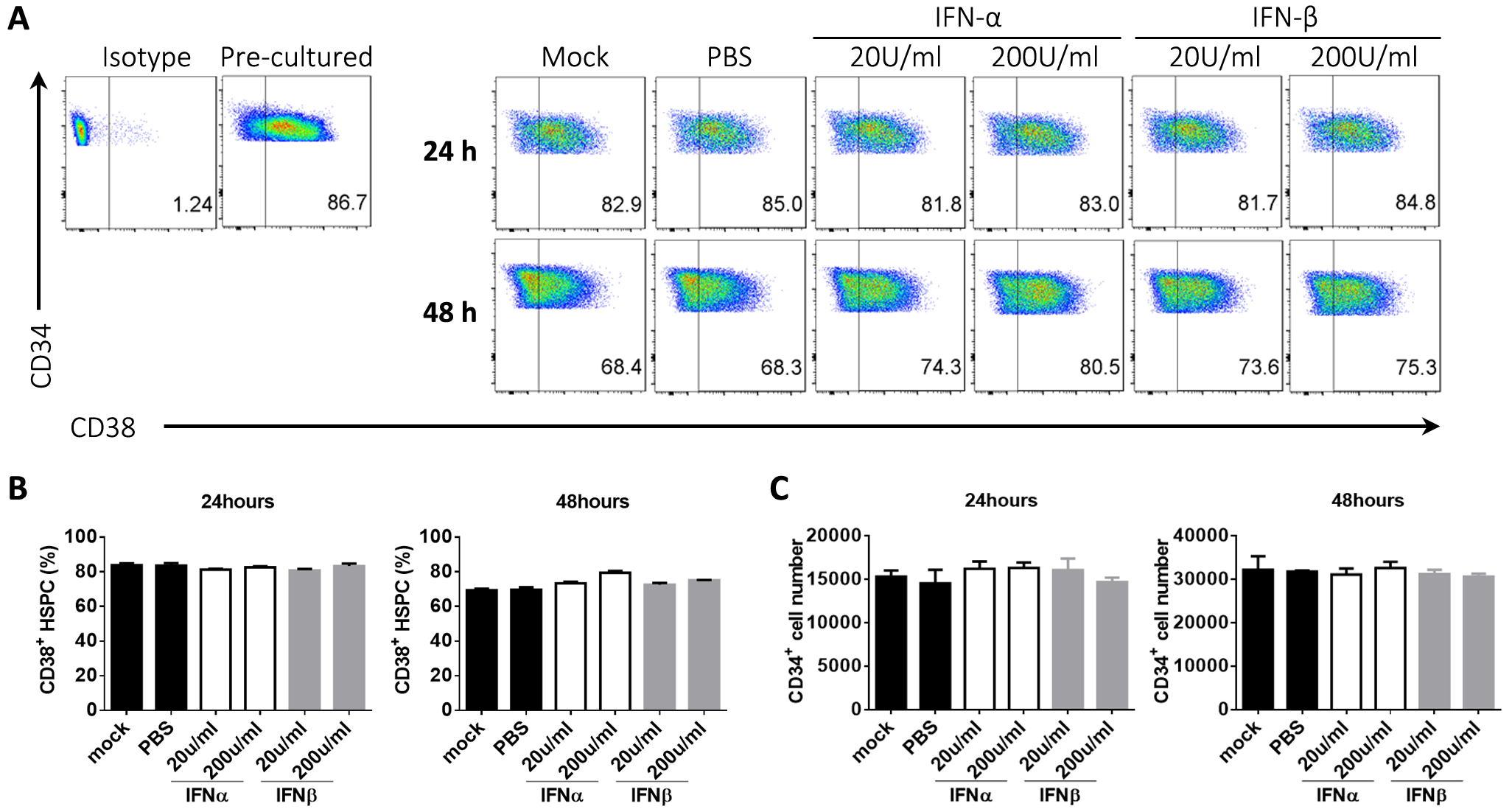

Supplement: S9 Fig — (A and B) Representative dot plots (A) and pool data (n = 3, B) indicated CD38 expression on purified CD34+ fetal liver-derived HPCs in vitro of 24- and 48-hours in the presence of IFN-α and IFN-β at 20 IU/ml and 200 IU/ml doses. The numbers in (A) indicated that the CD38 percentages on HPCs. * p < 0.05. (C) Pool data indicated the cell counts of CD34+ HPCs in vitro after 24- and 48-hour culture in the presence of IFN-I (n = 3). (TIF) [file ppat.1006505.s010.tif]

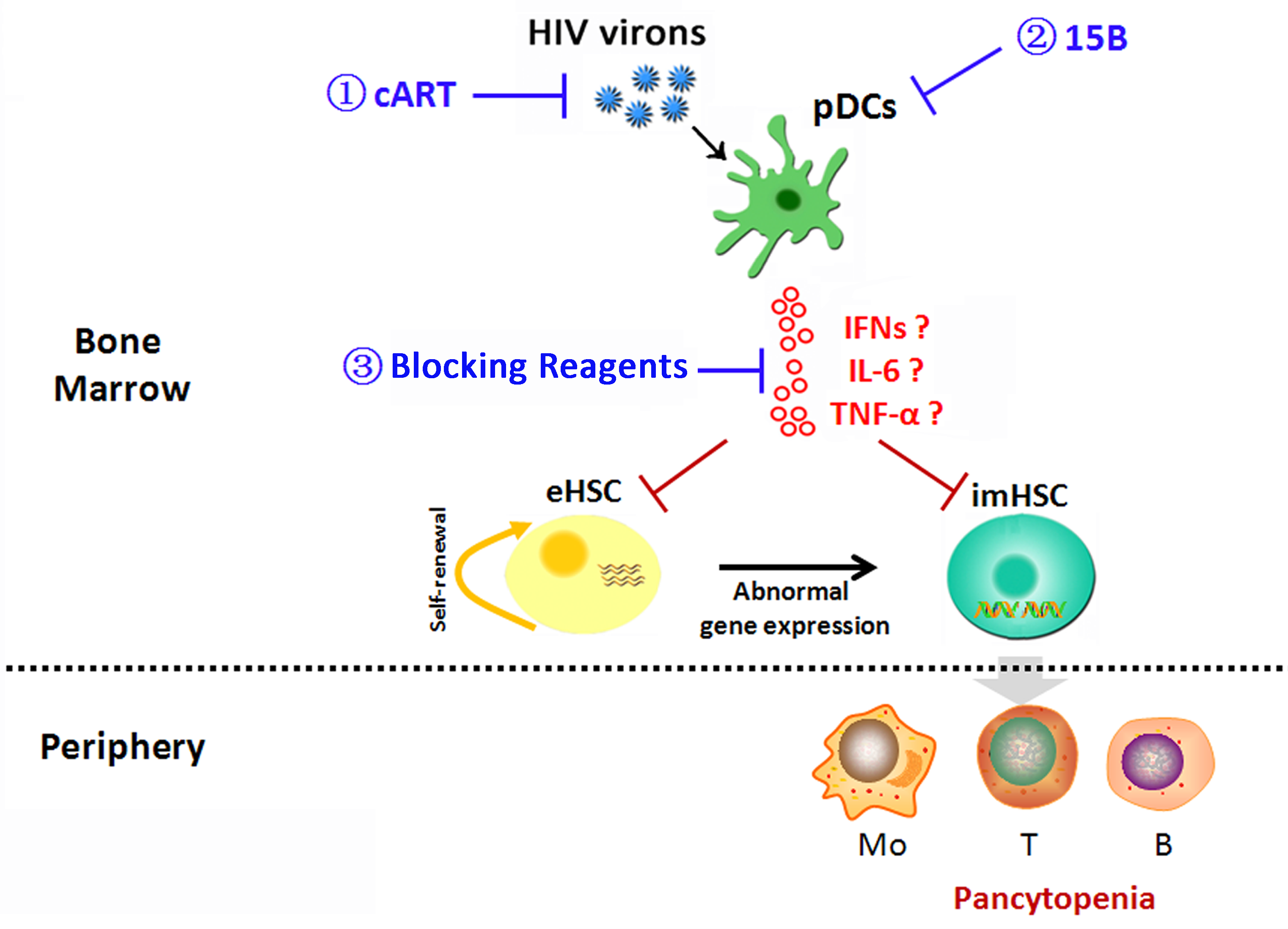

Supplement: S10 Fig — HIV-1 infection activates pDCs, possibly through type I IFNs, IL-6 and TNF-α, suppresses self-renewal proliferation of early CD34+CD38- HPCs and dysregulates gene expression profile in HPCs leading to their depletion and functional impairment. This effect on early HSCs subsequently contributes to HIV-1-induced pathogenesis such as loss of all human leukocyte cells (pancytopenia). Suppression of HIV-1 replication by ① cART, ② depletion of pDC or ③ blocking reagents against pDC-derived cytokines will restore HSC number and function. (TIF) [file ppat.1006505.s011.tif]
